# Microplastics bioaccumulation in fish: Its potential toxic effects on hematology, immune response, neurotoxicity, oxidative stress, growth, and reproductive dysfunction

**DOI:** 10.1016/j.toxrep.2024.101854

**Published:** 2024-12-09

**Authors:** Tapas Ghosh

**Affiliations:** a Department of Zoology, University of Kalyani, Nadia, Kalyani, West Bengal 741235, India; b Department of Zoology, Scottish Church College, Kolkata, West Bengal 700006, India

**Keywords:** Fish, Microplastic, Bioaccumulation, Reproductive dysfunction, Oxidative stress, Neurotoxicity

## Abstract

After being exposed, microplastics mostly bioaccumulated in guts and gills of fish, then, through circulation, spread and bioaccumulated in other tissues. Circulatory system of fish is impacted by the microplastic bioaccumulation in their tissues, influencing a number of hematological indices that are connected with immunity, osmotic pressure, blood clotting, molecular transport and fat metabolism. Variables like size, dose, duration, food consumption and species, all affect the bioaccumulation and toxicity of the microplastic, rather than the exposure routes. Microplastics lead to an imbalance in the generation of ROS and antioxidant defense of fish, which resulting in oxidative injury. Moreover, microplastics affect immunological responses through physico-chemical damage, hence produce neurotoxicity and modifies the activity of the acetylcholine esterase. Exposure to microplastics caused damage to the hepatic and gut tissue, affect intestinal barrier function and dysbiosis of microbial composition, altered the metabolism of host, affecting the activities of the digestive enzymes, eventually affecting the growth performance of fish. Microplastics exposure target the HPG axis and interfere with the process of steroidogenesis, apoptosis of the gonadal tissue, ultimately causing reproductive dysfunction. Fish exposed to microplastics have a range of toxic effects *viz.* alteration to immune, antioxidant and hematological indices, bioaccumulation, neurotoxicity, growth and reproductive dysfunction, all were examined in this present review by using different indicators.

## Highlights

• After being exposed, microplastics mostly bioaccumulated in guts and gills of fish, then, through circulation, spread and bioaccumulated in other tissues.
• Variables like size, dose, duration, food consumption and species, all affect the bioaccumulation and toxicity of the microplastic, rather than the exposure routes.
• Fish exposed to microplastics have a range of toxic effects *viz.* alteration to immune, antioxidant and hematological indices, bioaccumulation, neurotoxicity, growth and reproductive dysfunction.

## Graphical Abstract

**Figure:**
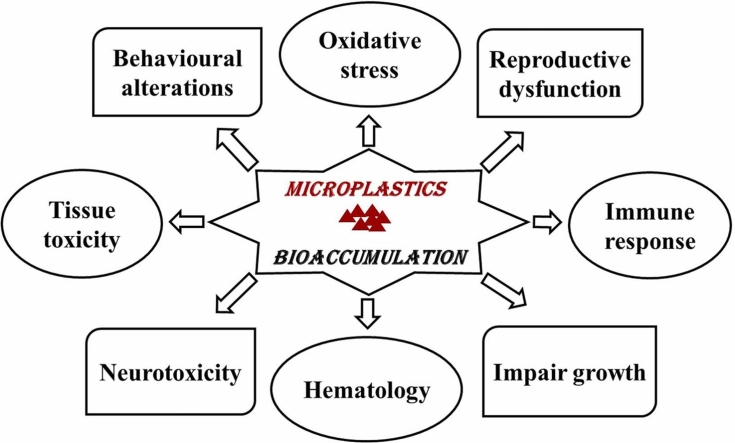

## 1 Introduction

Plastics are utilized in many aspects of our daily lives, including agriculture, industry, automobiles, construction, electrical products and electronics as well as apparel in addition to packaging [94]. Around 360 million tonnes of plastic were produced worldwide in the year 2018, out of which 80,000 tonnes are thought to have ended up in aquatic ecosystems. Around 10 % of the plastic waste produced worldwide is found in the marine ecosystems each year, amounting to 9.5 million tonnes of plastic waste, making it a significant hazardous material. Different types of plastics *viz.* polystyrene, polyethylene, polyethylene terephthalate, polyvinylchloride, ethylene vinyl acetate, polypropylene, and polyamide, are classified based on their structure and material used as well as having different properties and uses (Table 1). In aquatic habitat, microplastics, which is water insoluble, solid and small particles made of synthetic polymeric matrix components, breakdown from plastics and have an immediate effect on the aquatic life. Microplastics often have a mean size of less than 5 mm, however new research indicates that they should actually be categorized as varying from 1 to 1000 µm [54]. Worldwide, plastic pollution poses a serious threat to aquatic ecosystems. Microplastics have been found in both the marine as well as freshwater ecosystems, in sediments, in the column of water and along beaches across the seven continents. Microplastic can be found in the wild from a variety of sources, such as the decomposition of bigger plastic objects like drink or food containers, synthetic textile fibers, constituents of few cosmetic products and industrial effluents. Despite the fact that worries about how plastic pollution affects aquatic systems date back to 1978, both popular media and scientific literature have recently raised awareness of the issue, incorporating public remarks like restrictions prohibiting the utilization of microbeads in hygiene items. Data available on the potential threats of Microplastics on biota such as aquatic invertebrates and fish as well as aquatic environments, have been pushed for by concerned individual and researchers. As a result, the quantity of research looking at how Microplastics might affect aquatic food webs has risen substantially [26].

**Table 1:** A brief description of the characteristics of the microplastics [54].

**Type**	**Chemical formula**	**Melting point (ºC)**	**Glass transition temperature (ºC)**	**Specific gravity (g/cm**^**3**^**)**	**Item**
Polystyrene	(C_8_H_8_)n	210–249	90–100	1.01–1.09	Laboratory ware, facial cleanser, containers, utensils, food packaging
Polyethylene	(C_2_H_4_)n	105–130	−120	0.91–0.97	Motor oil bottles, plastic bags and film, toys, facial cleanser, paint, pipe, fiber, storage containers
Ethylene vinyl acetate	(C_2_H_4_)_m_(C_4_H_6_O_2_)_n_	80–120	−45–20	0.92–0.98	Agriculture films, photovoltaic modules, packaging, elastomeric materials, encapsulant
Polyvinylchloride	(C_2_H_3_Cl)_n_	100–260	82–85	1.16–1.30	Visors, shoe soles, containers, pipe, plastic film, insulation on electrical wires
Polypropylene	(C_3_H_6_)_n_	165	−20	0.90–0.91	Carpet backing, bumper, car seat, mat, bottle caps, netting, fishing gears, rope, strapping
Polyamide	(C_6_H_11_NO)_n_	220	47–50	1.13–1.15	Water, windshield wiper, fishing gears and nets, industrial cords, rope
Polyethylene terephthalate	(C_10_H_8_O_4_)_n_	260	70–80	1.34–1.39	Packaging, bottles, fiberfill, rope, textiles, strapping

There is evidence of Microplastics in the digestive tracts of aquatic invertebrates as well as fish that are caught from the wild; even in clams and mussels that were prepared for sale as food for humans [26]. Microplastic particles can be consumed by organisms in the aquatic ecosystems either actively, by creating confusion of possible prey or passively, at the time of filtration of the particles [19]. Research suggested that both the active and passive election can help species to avoid ingesting microplastics: *Acartia clause* and *Calanus helgolandicus* choose their prey based on smaller size compared to microplastics available in the system, and when food was available, the larvae of the *Tripneustes gratilla* chose microalgae over polyethylene beads. In contrast, *Eurytemora affinis* consumed latex beads more frequently than diatoms prey, and instead of eating sand, holothurian sea cucumbers consumed pieces of polyvinyl chloride and nylon, possibly because it was simpler for them to do so. Additionally, microplastic may inadvertently enter the body through absorption by the gills or attach itself to naturally occurring prey items like fish eggs or seaweed. Furthermore, ingested plastic particles may be soaked through the walls of the intestine [26].

Microplastics can persist in the gastrointestinal tract (GIT) of the aquatic fauna for days or even weeks after being swallowed or eaten, until they are eventually eliminated from the body by excretion. This retention period probably permits the migration of microplastics to new areas and/or microplastics moving up the food chain. When a single aquatic species is exposed to microplastics, it can have a detrimental effect on its ability to feed, survivability, reproductive performance as well as growth, because of things like clogged feeding mechanisms or decreased prey consumption. Nonetheless, it doesn’t seem that exposure to microplastics had the same consequences in different research. Certain organisms might be resistant to the stressors brought on by exposure to microplastics, and because it is to possible to egest the microplastics, cumulative effects may definitely be reduced. Furthermore, the shape of the microplastics *i.e.* spheres vs. fibers, may affect how easily they can be absorbed and how they affect an organism’s ability to perform. Hence, it is still challenging to forecast the total possible effect of microplastic pollution in aquatic environments [26].

In aquatic organisms, microplastic uptake and bioaccumulation in the GIT results in physical obstructions and inflammatory responses. Internal harm from such kind of damages includes ulcerative lesions and perforation of the gut. There is also a risk of stomach rupture and distortion, which can be fatal and resulting in death. Disruption of the endocrine system, reduced growth and reproduction, oxidative stress, cell lesions, metabolic disorders, genotoxicity, neurotransmission related disorder and reduced immunity are further negative effects that may result in death. Chemicals from different plastic additives, including colorants, flame retardants, UV filters and plasticizers such as bisphenol A and phthalates were used in the manufacturing process, are present in plastics. Additionally, when microplastics are moving through an aquatic ecosystem, organic pollutant *e.g*. metals, polycyclic aromatic hydrocarbons, polychlorinated biphenyls and organochlorine pesticides may be absorbed on their surface. Hence, microplastics can be chemically harmful to aquatic species with or without spike pollutants, causing endocrine disruption, liver stress, cellular injury, oxidative stress and metabolic alterations, resulting in the expression of cytochrome p450 1a (cyp1A) [54].

Because microplastics are small, they can be mistakenly consumed by aquatic species that misinterpret them as food, which allows them to enter the food web and have cumulative effects on predators further up the food chain via the process of biomagnification [6]. As a result, microplastics have an effect on fish in all stages, mollusks, crustaceans, seabirds, marine turtles and aquatic mammals. Fish are thought to be the most significant models for assessing the toxicity associated with microplastics since they are holding the uppermost predators and a typical representative of the aquatic environments. As a result, microplastic consumption not only harms the aquatic ecology but at the same time jeopardizes food safety because fish are a significant contributor of first class protein for human populations and fish protein holds the superior position among all the animal based dietary protein sources because of their easy affordability, digestibility as well as availability, provided the fact that animal based proteins are termed as ‘first class’ because they contain all the essential amino acids [27], [54]. Hence, in order to provide a general indicators of the toxicity generated by microplastics, this review examined the current literature on microplastics in aquatic environments. This goal was accomplished by analyzing the effects of microplastic exposure on bioaccumulation, alteration of the hematological profiles, immune response, neurotoxicity, oxidative stress, growth, reproductive performance and particular gene expressions in fish.

## 2 Bioaccumulation of the microplastic

In aquatic ecosystems, plastic waste breaks down into tiny fragments as a result of physico-chemical reactions with waves, sunlight and organisms present in the system. Aquatic species may unintentionally consume materials that break down to the microplastic level as food [64]. Specifically elevated level of microplastics are seen in estuarine and marine ecosystems, where both marine and freshwater fish are acquiring different microplastics [5]. When microplastic particles build up in the body, they travel through the cells and enter the lymphatic or circulatory systems, where they are distributed across the body. Because microplastic consumption degrades the GIT both functionally as well as structurally, it can affect the growth and nutrition of fish [94]. Microplastics often gather in the GIT of fish but also carried to liver along with other organs. Finding the main microplastics that build up in the fish organs after microplastics exposure is crucial for identifying organs that are highly toxic to microplastics and assessing the underlying mechanisms [49]. The immune system, growth, energy metabolism, oxidative stress and several other biomarkers can all be adversely affected by tissue buildup of micro- and nano-plastics. Fish can absorb microplastic through their mouths, skin or gills, and it can quickly build up in their gills and guts [11]. When fish are exposed to microplastic for an extended period of time, the intestines become inflamed, which can cause harm and imbalances the gut microbiota, ultimately leading to metabolic disorders as well as diseases [51].

Microplastics that come into the GIT of fish can be eliminated in their urine and feces as well as in other tissues, whereby microplastics are transported via the lining of gut epithelium. Microplastics are primarily stored in the adipose tissues of animals after being absorbed into the gut, where they can be taken into the tissues in the gut anaerobic microenvironment. Bioaccumulation of the microplastics is impacted by the exposure route *e.g.* dietary or water borne and aquatic ecosystem *e.g.* marine or freshwater. In order to reduce the loss of ion in freshwater condition with lower osmotic pressure, gills of the freshwater fish contain chloride cells that absorb ions actively from the surrounding water and excrete significant amounts of water through urine. On the other hand, in marine ecosystems having higher osmotic pressure, marine fish drink an enormous amount of water to stay hydrated. Because of this, when marine fish are exposed to the same dose of microplastics, their accumulation is larger due to these physiological differences [54]. A number of other variables *viz.* dose and duration of the exposure, particle size, food consumption and fish species, all can impact the bioaccumulation of the microplastic [22]. Specifically, a significant determinant of microplastic particle accumulation in fish is their size. For example, the size of the microplastics influences how much the circulatory system transports them, which leads to variations in the bioaccumulation of the microplastic in the tissues [54]. Microplastics smaller than 5 µm have the ability to transform, get past the gut cells, get into the bloodstream and migrate to the tissues [50].

Table 2 displays the bioaccumulative profile of fish after microplastics exposure according to various exposure pathways *viz.* marine vs freshwater and dietary vs waterborne exposure. According to Ding et al. [22], *Oreochromis niloticus* underwent considerable bioaccumulation after being exposed to polystyrene microplastics (size: 0.1 µM). More microplastics are specifically accumulated in gills and gut than in the brain and liver. The buildup of polystyrene in the colon spreads to the hemolymph and can then use the circulatory system to spread to the other tissues. Exposure to microplastic can induce damage to the gut that results in cell proliferation and villi cracking. It can also change the concentration of ions *i.e.* calcium, in the gut, which can affect the function of the gut [56]. Furthermore, the absorptive pathway demonstrates that aquatic particles can be orally transferred to water-gills-circulation-tissues as well as ingestion-gut-circulation-tissues. Polystyrene having 0.1 mm size can potentially enter the bloodstream and get into the brain tissue. Bioaccumulation and the size of the microplastic are tightly connected. For example, larger particles support the balance of bioaccumulation, whereas smaller microplastics tend to stay in tissues for longer periods of time in fish [76]. Gills, a vital organ, which helps in respiration that govern acid bases and ions and are immediately exposured with numerous hazardous compounds in the aquatic ecosystem via interaction with water. As a consequence, gills constitute a key channel for the entrance of several contaminants to the tissue of fish [78]. The most significant determinant in deciding accumulation after being exposed to harmful toxicants is the exposure route, however, in case of microplastic, size of the particle was more significant in determining the propensity of the bioaccumulation than the route of the exposure. Additionally, depending on the particle size, bioaccumulation of the microplastic in the brain tissue can directly poses toxic effects to central nervous system (CNS), allowing microplastics to cross the blood-brain barrier and accumulate in the brain. The bioaccumulation of the microplastics exhibiting toxicity and damages the tissues, making it impossible to operate normally. Because gut tissue plays a crucial role in digestion as well as absorption, feeding activity exposes it to polluted substances directly, increasing microplastic level and causing bioaccumulation of the toxicant. The kidneys and liver are exposed to toxic chemicals during their function in detoxification as well as excretion of the particles from circulation, which may result in a large accumulation of the microplastic [54]. According to reports, microplastics first gather in the head and yolk sac of *Danio rerio* subjected to polystyrene nanoplastic, subsequent bioaccumulation occurred in many tissues such as the GIT, pancreas, gall bladder, liver and pericardium. Elevated concentration in the yolk sac suggests that metabolic disorders may be caused by polystyrene. When exposed to polystyrene nanoplastic, the pericardium experiences considerable microplastic bioaccumulation and a marked decrease in heart rate, which may result in cardiac toxicity [70].

**Table 2:** Bioaccumulative figure of the microplastics in fish exposed to dietary vs waterborne as well as fresh vs saltwater.

**Mode of exposure**		**Applied species**	**MPs type**	**size**	**Dose**	**Duration**	**Bioaccumulation pattern**	**references**
**Freshwater**	**Dietary**	*Carassius auratus*	Polyethylene, polyester fiber	63–250 µm	50 particles/pellet	1.5, 4, 8, 16, 32, 48, 96, 144 hours	No accumulation	[32]
			Ethylene vinyl acetate	0.7–5.0 mm	55–76 particles/pellet	6 weeks	Bioaccumulation in gills and GIT	[48]
			Polyethylene acetate	4.9–5.0 mm	15 particles/pellet	6 weeks	No accumulation	
			Polystyrene	2.5–3.0 mm	15 particles/pellet	6 weeks	No accumulation	
	**Waterborne**	*Danio rerio*	Green fluorescent polystyrene	20–100 nm	0.1, 1 and 10 ppm	Embryo (6, 24 hours), Larvae (48,72, 120 hours)	Yolk sac and head	[70]
			Green fluorescent polystyrene	5, 20 µm	20 mg/L	4 h, 12 h, 1 day, 2 days, 7 days	Gut, liver, gills (5 µm); intestine, gills (20 µm)	[61]
			Polystyrene	150 µm	75 mg/L	12, 24 days	gut	[103]
			Fluorescent polystyrene	0.1, 20 µm	2 mg/L	0, 4, 12, 24, 72 hours	Intestine > liver > gill (0.1 µm) and intestine (20 µm)	[74]
			Polystyrene	50 nm	1 mg/L	1, 2, 3 days	Viscera > gill > head > muscle	[17]
			Green fluorescent polystyrene	70 nm	0.5, 1.5, 5 ppm	30 days	Gonad > gut = brain > liver	[81]
		*Paramisgurnus dabryanus*	Polyethylene	8–12 µm	1, 5, 10 mg/L	21 days	Intestine, blood cells	[100]
		*Pseudobagrus fulvidraco*	Polyethylene	0.49–92 µm	100, 200, 5000 and 10,000 mg/L	96 hours	Intestine ˃ gills ˃ liver	[55]
		*Carassius carassius*	Polyamide	-	4, 8, 16, 32 and 64 mg/L	14 days	Gut ˃ gills ˃ liver	[18]
		*Oreochromis niloticus*	Green fluorescent polystyrene	0.3, 5, 70–90 µm	100 µg/L	6 days	Intestine > brain > liver > gills (0.3 µm); gills > brain > liver > intestine (5 µm); Liver > brain > gill > intestine (70–90 µm)	[21]
						14 days	Intestine > gills > liver > brain (0.3 µm); intestine > liver > brain > gills(5 µm); intestine > liver > gill > brain (70–90 µm)	
			Green fluorescent polystyrene	0.1 µm	1, 10, 100 µg/L	14 days	Intestine > gill > liver = brain	[22]
			Polystyrene	1 µm	0.01, 0.1 and 1 mg/L	14 days	Gut, Gills, spleen, muscle, liver, brain and gonad	[20]
		*Oryzias latipes*	Fluorescent polystyrene	39.4 nm	10 mg/L	7 days	Gall bladder > gut > ovary > gill > testis > kidney > liver > brain	[53]
		*Carassius carassius*	Polystyrene	53, 180 nm	0.1 g/L	67 days	Brain	[64]
			Polyethylene	22–71 µm	0, 4, 8, 16, 32 and 64 mg/L	14 days	Gut ˃ gills ˃ liver	[110]
**Seawater**	**Dietary**							
		*Sparus aurata*	Polyvinylchloride, polystyrene, polyamide, polyethylene	75 µm	2800 particles/day	45 days	Liver (less than one particle per liver)	[50]
	**Waterborne**							
		*Mugil cephalus*	Polystyrene, polyethylene	0.01–0.1, 0.1–0.5, 0.5–1.0, 1.0–5.0 mm	0.03375 g/L	7 days	Gut > liver	[7]
		*Sebastes schlegelii*	Green fluroscent polystyrene	15 µm	1 × 10^6^ microspheres/L	21 days	Gut, gill	[107]
		*Oryzia melastigma*	Polystyrene	10 µm	2, 20, 200 µg/L	60 days	Gills > gut > liver	[96]

In general, the size of the microplastic influences both the tendency of bioaccumulation in tissue as well as the potential toxicity during the fragmentation process. According to investigation, polystyrene microplastic size *i.e.* 0.3, 5 and 70–90 µm have an impact on how they accumulate in fish. It was found that the largest bioaccumulation happens in organs following a 6 day exposure of the gut to 0.3 µm, the gills to 5 µm as well as the liver to 70–90 µm particles in *Oreochromis niloticus*. Though, the degree of bioaccumulation varied after two weeks of exposure, with gut tissue exhibiting the greatest accumulation for all particle sizes. Hence, the particle size of the microplastics can affect how the primary bioaccumulation structure appears, and the duration of the exposure can affect the degree of bioaccumulation occurs when the particles are disseminated throughout the circulatory system. Smaller microplastic particles were observed in the tissue of the gut of the *O. niloticus* group subjected for bigger particles (70–90 µm) exposure, consequently, big particles may be susceptible to intestinal fragmentation during the process of digestion. By changing the bioavailability of the polystyrene, particle fragmentation following fish ingestion may enhance the toxicological potency of the microplastic, hence the potential threat of the large size microplastics cannot be ruled out and should have taken into consideration [21]. Compared to 20 µm polystyrene microplastics, the accumulation of the 5 µm polystyrene in tissue was two times higher in *D. rerio*. Due to its higher specific surface area than the other tissues, the gut may be more prone to toxicity for the same amount of small sized particles. Furthermore, while 20 µm polystyrene do not bioaccumulate in the liver, but 5 µm particles do. Size dependent variations in metastasis to the circulation may be the cause of this phenomena [61]. After being exposed to microplastics, the occurrence of bioaccumulation was observed in gut and gills of *Dicentrarchus labrax*, which may later be absorbed by the epithelial cells by the process of endocytosis, permitting it to enter the internal organs via the circulation or pass through biological barriers. The most important determinant of microplastic bioaccumulation in fish after exposure is thought to be their size. Specifically, exposure to microplastics that are nanoscale results in a more varied bioaccumulation in the tissue of fish [116]. The exposure of nano-scale polystyrene (size: 50 nm) exhibited that reduction in size of the plastic particle, more tissues such as CNS, brain and several other tissues also were penetrated [16], [17]. It has been established that nanoplastics of 53 and 180 nm polystyrene can proceed through the blood-brain barrier and aggregate in the brain tissue of *Carassius carassius* [64]. Nanoplastic polystyrene particles of 39.4 nm size after passing via the gills or gut, entering into the circulation and bioaccumulate in several tissues such as liver, kidney, ovary, testis, gut, gall bladder and gills of *Oryzias latipes*. Nanoparticles can cross the blood-brain barrier and enter the brain, despite the fact that the bioaccumulation in the brain tissue is low [53]. Microplastics were detected in the captured fish, *Lateolabrax maculatus* for commercial purpose and the maximum bioaccumulation was seen in intestine followed by gills, whereas muscle and liver tissues contain no microplastic. Authors also suggested that microplastic found in the gills were smaller than microplastics found in the intestines [87]. Yu et al. [110] recommended that there were no toxicological interplays between microplastics polyethylene and duration of the exposure in *Carassius carassius* because the interplay between these two did not produce any significant impacts.

The bioaccumulation of the microplastics in fish gills and guts after exposure suggests that ingestion and absorption are the main routes of microplastic absorption. Furthermore, because of their vast surface area and exposure to water, microplastics can bioaccumulate in huge amounts in the gills. The microplastics found in the body of fish have the ability to be absorbed across gut barrier and cells, and stay toxic in the tissues for extended period of time. However, microplastics that are consumed in the digestive system have the ability to excrete to the bottom of the aquatic ecosystem, where they could generate potential threats to the benthic aquatic organisms [107]. Size of the particle plays a major role in microplastic bioaccumulation, with smaller ones accumulating more readily, independent of the mode of absorption [116]. Conversely, it is more likely for larger microplastics to stay in the liver than the smaller ones, which can be easily eliminated by the liver via circulation [50]. The retention of 6 different types of polymers (nylon, high-density polyethylene, ethylene vinyl acetate, nitrile, polypropylene and polystyrene) in the edible tissue *i.e.* flesh of the *Labeo bata*, *Catla catla* and *Oreochromis mossambicus*, out of the 43 total fish species were reported in the natural lake environment. The greatest concentration of microplastics was found in the flesh of the *L. bata* [66]. Hence, it is very obvious that, due to their food habit of eating fish in diet, definitely there is a chance of contamination and threats to the human civilization is under scrutiny of the risks associated with the microplastics. Microplastics tend to bioaccumulate in fish in accordance to the size of the microplastic, it is therefore required to determine the target organ through further investigation [54].

## 3 Effect of microplastic on fish health

Microplastic uptake and bioaccumulation in the GIT of the aquatic fauna results in physical obstructions and inflammatory responses. Internal harm from such kind of damages includes ulcerative lesions and perforation of the gut. There is also a risk of stomach rupture and distortion, which can be fatal and resulting in death. Disruption of the endocrine system, reduced growth and reproduction, oxidative stress, cell lesions, metabolic disorders, genotoxicity, neurotransmission related disorder and reduced immunity are further negative effects that may result in death. Chemicals from different plastic additives, including colorants, flame retardants, UV filters and plasticizers such as bisphenol A and phthalates were used in the manufacturing process, are present in plastics. Additionally, when microplastics are moving through an aquatic ecosystem, organic pollutant such as metals, polycyclic aromatic hydrocarbons, organochlorine pesticides and polychlorinated biphenyls may be absorbed on their surface, and that can have a detrimental consequences on the health of aquatic fauna either in presence or absence of spike pollutant [54]. The following part of the article discussed about the potential toxicity caused by the microplastic exposure in fish.

### 3.1 Effect on blood profile

Toxic chemicals entering the aquatic ecosystem have an impact on the water quality parameters, and it may result in modification of blood profiling of fish. Exposure of toxic chemical alters the ability of blood to carry oxygen and electrolyte balance, which reduces cell size because of exosmosis of the erythrocytes. After exposure to infection, chemical toxicity and different stressors, hematological profile *viz.* WBCs, RBCs, hemoglobin, hematocrit, total protein, glucose, alkaline phosphatase (ALP), cholesterol, alanine aminotransferase (ALT) and aspartate aminotransferase (AST), are crucial markers for assessing the status of the fish health. When microplastic penetrates the circulatory system of fish, they can result in fatal reactions and metabolic disorders like oxidative stress, gene expression, endocrine abnormalities and immunological responses. 1–4 % of the microplastic particles that are absorbed into the fish gut are thought to make their way into the circulation, where they can trigger the tissue allergic reactions or localized inflammation due to their size (either micro or nano). Microplastics ≤ 5 µm have the ability to penetrate the bloodstream and hematological profile such as hemoglobin, hematocrit and RBCs, leading to inflammatory responses, metabolic disorders and lipid peroxidation. Exposure with toxic chemicals generally cause destruction and hemolysis of the erythrocytes, which lowers hematological profile, eventually leading to anemia. The deleterious effects of the toxicity may be the cause of the decrease in hemoglobin level and RBC count brought on by exposure to harmful chemicals, and a drop in hemoglobin level raises the possibility of tissue function being harmed since there is not enough oxygen supply. Exposure to microplastic can induce physico-chemical damage to the erythrocytes membranes, which can result in hemolysis and affect hemoglobin concentrations as well as hematocrit levels [54].

Elevated level of triglycerides and cholesterol may be caused by metabolic abnormalities and biosynthesis of lipoproteins and lipids in the liver of fish. Additionally, microplastics exposure may results in injury of cellular membrane, modifications of transmembrane gradients, inhibition or stimulation of the enzymes and alterations in hormone content associated with lipid metabolism, causing modifications in the levels of cholesterol and triglyceride [8]. Fish metabolism may be impacted by microplastic consumption due to changes in blood cholesterol and triglyceride ratios as well as changes in the distribution of cholesterol in liver and muscle tissue [49]. In order to consistently maintain the osmotic equilibrium and stop fluid to leak from circulation, plasma protein is essential in the circulatory system [23], especially albumin, because it act as a carrier molecule for drug and medicine as well as determines the overall pharmacokinetic as well as pharmacodynamic profile of the drugs [29], [28]. When microplastics get into the circulatory system of fish, they can combine with blood proteins like globulin and albumin to create plastic-protein complexes that could affect circulation of blood [109]. Microplastic polyvinylchloride exposure resulted in damage to RBC generation and suppression of hemoglobin biosynthesis, which is why *Clarias gariepinus*, displayed a progressive decline in hemoglobin level in a time specific pattern and the suppression of the biosynthesis might be due to harm to erythropoiesis. When maturation of the erythrocyte is disrupted and alteration of the status of macrocytic or microcytic anemia is happened, it indicates a decline in hematological profile brought on by the exposure to microplastic [47]. Hence, exposure of the microplastic in fish reduced the amount and synthesis of hemoglobin generated by stress, and it might have caused tissue oxygenation dysfunction, which would have made it toxic [54]. Microplastics that are absorbed by fish diffuse across the cellular membrane and enter the circulation, bioaccumulated in the body and hence, blood profiling could therefore be utilized as a sensitive biomarker of microplastic exposure [83]. The hematological profiles of the *O. niloticus* such as hemoglobin, hematocrit and RBCs altered after being exposed to microplastic. This phenomena resulted in hemolysis brought on by microplastic toxicity and hemodilution of blood after tissue injury [35]. Table 3 displays the impact of microplastics on blood profile of fish according to various exposure pathways (Dietary vs waterborne and sea vs freshwater).

**Table 3:** Blood profiles of the fish after exposured with microplastic particles.

**Route of exposure**	**Applied organism**	**Microplastics type**	**Microplastics size**	**Dose**	**Duration**	**Response dose**	**Response**	**Reference**
**ALP (µ/L)**								
**Saltwater**								
**Dietary**								
	Sparus aurata	Polystyrene	1–20 µm	25 and 250 mg/kg	21 days	Both	↑	[69]
**Waterborne**	*Symphysodon aequifasciatus*	Polyethylene	70–88 µm	200 µg/L (28 and 31ºC)	30 days	200 µg/L (28 and 31ºC)	↓	[97]
	*Sparus aurata*	Polymethylmethacrylate	40 nm	0.0001, 0.01, 0.1, 1 and 10 mg/L	24 and 96 hours	0.0001, 0.01, 0.1, 1 and 10 mg/L	→	[12]
	*Dicentrarchus labrax*	Polymethylmethacrylate	45 nm	0.02, 0.2 and 2 mg/L	4 days	0.2 and 2 mg/L	↓	[13]
**Freshwater**								
**Waterborne**	*Cyprinus carpio*	Polyethylene	-	250 and 500 µg/L	30 days	Both	↑	[8]
		Polyvinylchloride	100–200 µm	10, 20 and 30 %	60 days	20 and 30 %	↑	[60]
		Polyethylene	-	175, 350, 700 and 1400 µg/L	30 days	175, 350, 700 and 1400 µg/L	↑	[9]
	*Pseudobagrus fulvidraco*	Polyethylene	0.49–92 µm	100, 200, 5000 and 10,000 mg/L	96 hours	˃5000 mg/L or 10,000 mg/L	↑	[55]
	*Oreochromis niloticus*	Microplastics	˃100 nm	1, 10 and 100 mg/L	15 days	1, 10 and 100 mg/L	↑	[35]
		Polystyrene	1 µm	0.01, 0.1 and 1 mg/L	14 days	0.1 and 1 mg/L	↑	[20]
**ALT**								
**Saltwater**								
**Dietary**	*Sparus aurata*	Medium density polyethylene/ Ultra-high molecular weight polyethylene	75 µm	0.1 g/kg	45 days	-	→	[50]
		Polyvinylchloride high molecular weight	75 µm	0.1 g/kg	45 days	-	→	
		Polyvinylchloride low molecular weight/ Polystyrene/ Polyamide	75 µm	0.1 g/kg	45 days	-	→	
**Waterborne**	*Sparus aurata*	Polymethylmethacrylate	40 nm	0.001, 0.01, 0.1, 1 and 10 mg/L	24 and 96 hours	-	→	[12]
	*Dicentrarchus labrax*	Polymethylmethacrylate	45 nm	0.02, 0.2 and 2.0 mg/L	4days	-	→	[13]
**Freshwater**								
**Waterborne**	*Danio rerio*	Polystyrene	100 nm	10 and 100 µg/L	35 days	Both	↑	[91]
	*Pseudobagrus fulvidraco*	Polyethylene	0.49–92 µm	100, 200, 5000 and 10,000 mg/L	96 hours	˃5000 mg/L or 10,000 mg/L	↑	[55]
	*Cyprinus carpio*	Polyethylene	-	250 and 500 µg/L	30 days	250 and 500 µg/L	↑	[8]
		Microplastics	-	1 and 2 mg/L	21 days	-	→	[34]
		Polyethylene	-	175, 350, 700 and 1400 µg/L	30 days	175, 350, 700 and 1400 µg/L	↑	[9]
	*Oreochromis niloticus*	Microplastics	˃100 nm	1, 10 and 100 mg/L	15 days	1, 10 and 100 mg/L	↑	[35]
		Polystyrene	1 µm	0.01, 0.1 and 1 mg/L	14 days	0.1 and 1 mg/L	↑	[20]
**AST (µ/L)**								
**Saltwater**								
**Dietary**	*Sparus aurata*	Polymethylmethacrylate	40 nm	0.1 g/kg	45 days	-	→	[50]
**Waterborne**		Polymethylmethacrylate	40 nm	0.001, 0.01, 0.1, 1 and 10 mg/L	24 and 96 hours	-	→	[12]
	*Dicentrarchus labrax*	Polymethylmethacrylate	45 nm	0.02, 0.2 and 2.0 mg/L	4 days	-	→	[13]
	*Cyprinus carpio*	Polyethylene	-	175, 350, 700 and 1400 µg/L	30 days	175, 350, 700 and 1400 µg/L	↑	[9]
**Freshwater**								
**Waterborne**	*Oreochromis niloticus*	Microplastics	˃100 nm	1, 10 and 100 mg/L	15 days	10 and 100 mg/L	↑	[35]
		Polystyrene	1 µm	0.01, 0.1 and 1 mg/L	14 days	0.1 and 1 mg/L	↑	[20]
	*Danio rerio*	Polystyrene	100 nm	10 and 100 µg/L	35 days	Both	↑	[91]
	*Cyprinus carpio*	Polyethylene	-	250 and 500 µg/L	30 days	-	→	[8]
		Microplastics	-	1 and 2 mg/L	21 days	-	→	[34]
		Polyethylene	-	175, 350, 700 and 1400 µg/L	30 days	175, 350, 700 and 1400 µg/L	↑	[9]
**Cholesterol (mg/L)**								
**Saltwater**								
**Waterborne**	*Sparus aurata*	Polymethylmethacrylate	40 nm	0.001, 0.01, 0.1, 1 and 10 mg/L	24 hours	0.001, 0.01, 0.1, 10 mg/L	↓	[12]
					96 hours	0.001, 0.01. 0.1, 1 and 10 mg/L	↓	
	*Dicentrarchus labrax*	Polymethylmethacrylate	45 nm	0.02, 0.2 and 2.0 mg/L	4 days	-	→	[13]
**Freshwater**								
**Dietary**	*Carassius carassius*	Polystyrene	28 nm	0.01 % in bottles	14 days	-	→	[15]
	*Clarias gariepinus*	Low density polyethylene	˂60 µm	50 and 500 µg/L	96 hours	Both	↓	[52]
**Waterborne**	*Cyprinus carpio*	Polyethylene	-	250 and 500 µg/L	30 days	Both	↑	[8]
		Polyethylene	-	175, 350, 700 and 1400 µg/L	30 days	175, 350, 700 and 1400 µg/L	↑	[9]
	*Oreochromis niloticus*	Microplastics	˃100 nm	1, 10 and 100 mg/L	15 days	1, 10 and 100 mg/L	↑	[35]
**Glucose (mg/dL)**								
**Saltwater**								
**Dietary**	*Sparus aurata*	*Polyvinylchloride*	40–150 µm	100 and 500 mg/kg	15 days	500 mg/kg	↓	[23]
					30 days	-	→	
**Waterborne**	*Sparus aurata*	Polymethylmethacrylate	40 nm	0.001, 0.01, 0.1, 1 and 10 mg/L	24 and 96 hours	-	→	[12]
	*Dicentrarchus labrax*	Polymethylmethacrylate	45 nm	0.02, 0.2 and 2.0 mg/L	4 days	-	→	[13]
**Freshwater**								
**Waterborne**	*Prochilodus lineatus*	Polyethylene	10–90 µm	20 µg/L	24 and 96 hours	-	→	[78]
	*Cyprinus carpio*	Polyethylene	-	250 and 500 µg/L	30 days	Both	↑	[8]
		Polyethylene	-	175, 350, 700 and 1400 µg/L	30 days	175, 350, 700 and 1400 µg/L	↑	[9]
		Microplastics	-	1 and 2 mg/L	21 days	-	→	[34]
	*Oreochromis niloticus*	Microplastics	˃100 nm	1, 10 and 100 mg/L	15 days	1, 10 and 100 mg/L	↑	[35]
		Polyamide	500 µm−4 mm	10 mg/L	15 days	10 mg/L (30, 33 and 36ºC)	↓	[38]
		Polystyrene	1 µm	0.01, 0.1 and 1 mg/L	14 days	0.1 and 1 mg/L	↑	[20]
**Total protein (g/dL)**								
**Saltwater**								
**Dietary**	*Sparus aurata*	Polyvinylchloride	40–150 µm	100 and 500 mg/kg	15 and 30 days	-	→	[23]
**Freshwater**								
**Waterborne**	*Clarias gariepinus*	Low density polyethylene	˂60 µm	50 and 500 µg/L	96 hours	500 µg/L	↑	[52]
	*Cyprinus carpio*	Polyethylene	-	250 and 500 µg/L	30 days	Both	↑	[8]
		Microplastics	-	1 and 2 mg/L	21 days	2 mg/L	↓	[34]
		Polyethylene	-	175, 350, 700 and 1400 µg/L	30 days	175, 350, 700 and 1400 µg/L	↓	[9]
		Polypropylene	0.9085 ± 0.0312 mm	1 and 2.5 g/L	1–7 days		↓	[106]
	*Oreochromis niloticus*	Microplastics	˃100 nm	1, 10 and 100 mg/L	15 days	100 mg/L	↑	[35]
		Polystyrene	1 µm	0.01, 0.1 and 1 mg/L	14 days	0.1 and 1 mg/L	↑	[20]
**Dietary**	*Cyprinus carpio*	Polypropylene	0.9085 ± 0.0312 mm	100 and 250 mg/g	1–7 days		↓	[106]
**RBCs (Million/mm**^**3**^**)**								
**Freshwater**								
**Dietary**	*Clarias gariepinus*	Polyvinylchloride	100 µm	0.5, 1.5, 3 % in pellet	45 days	-	→	[47]
	*Pseudobagrus fulvidraco*	Polyethylene	0.49–92 µm	100, 200, 5000 and 10,000 mg/L	96 hours	˃5000 mg/L	↓	[55]
	*Prochilodus lineatus*	Polyethylene	10–90 µm	20 µg/L	24 and 96 hours	-	→	[78]
	*Oreochromis niloticus*	Microplastics	˃100 nm	1, 10 and 100 mg/L	15 days	1, 10 and 100 mg/L	↓	[35]
		Polystyrene	1 µm	0.01, 0.1 and 1 mg/L	14 days	0.1 and 1 mg/L	↓	[20]
**Waterborne**	*Carassius carrasius*	Polyethylene	22–71 µm	4, 8, 16, 32 and 64 mg/L	14 days	≥ 32 mg/L	↓	[110]
		Polyamide	-	4, 8, 16, 32 and 64 mg/L	14 days	-	↓	[18]
	*Oreochromis niloticus*	Polyamide	500 µm−4 mm	10 mg/L	15 days	10 mg/L (30, 33 and 36ºC)	↓	[38]
**Hemoglobin (g/dL)**								
**Freshwater**								
**Dietary**	*Clarias gariepinus*	Polyvinylchloride	100 µm	0.5, 1.5, 3 % in pellet	45 days	1.5 % in pellet	↓	[47]
**Waterborne**	*Prochilodus lineatus*	Polyethylene	10–90 µm	20 µg/L	24 and 96 hours	-	→	[78]
	*Pseudobagrus fulvidraco*	Polyethylene	0.49–92 µm	100, 200, 5000 and 10,000 mg/L	96 hours	˃5000 mg/L	↓	[55]
	*Oreochromis niloticus*	Microplastics	˃100 nm	1, 10 and 100 mg/L	15 days	1, 10 and 100 mg/L	↓	[35]
		Polyamide	500 µm−4 mm	10 mg/L	15 days	10 mg/L (30, 33 and 36ºC)	↑	[38]
		Polystyrene	1 µm	0.01, 0.1 and 1 mg/L	14 days	0.1 and 1 mg/L	↓	[20]
	*Carassius carrasius*	Polyethylene	22–71 µm	4, 8, 16, 32 and 64 mg/L	14 days	≥ 32 mg/L	↓	[110]
		Polyamide	-	4, 8, 16, 32 and 64 mg/L	14 days	-	↓	[18]
**Hematocrit (PCV) (%)**								
**Freshwater**								
**Waterborne**	*Prochilodus lineatus*	Polyethylene	10–90 µm	20 µg/L	24 and 96 hours	-	→	[78]
	*Pseudobagrus fulvidraco*	Polyethylene	0.49–92 µm	100, 200, 5000 and 10,000 mg/L	96 hours	˃5000 mg/L	↓	[55]
	*Oreochromis niloticus*	Microplastics	˃100 nm	1, 10 and 100 mg/L	15 days	100 mg/L	↓	[35]
		Polystyrene	1 µm	0.01, 0.1 and 1 mg/L	14 days	0.1 and 1 mg/L	↓	[20]
	*Carassius carrasius*	Polyethylene	22–71 µm	4, 8, 16, 32 and 64 mg/L	14 days	≥ 32 mg/L	↓	[110]
		Polyamide	-	4, 8, 16, 32 and 64 mg/L	14 days	-	↓	[18]

↑: increase in response, →: no response, ↓: decrease in response

Maintaining the homeostasis of glucose involves balancing the generation with its storage as glycogen [78]. It was revealed that a noteworthy rise in total protein, cholesterol and glucose in *O. niloticus* treated with microplastics [35]. According to Banaee et al. [8], exposure to microplastic markedly enhanced the total protein, cholesterol and glucose level of *Cyprinus carpio*. The impact of plasticizers on glucose metabolism and resistance to insulin was linked to the elevated level of glucose [78]. The glucose levels of fish that are altered by microplastic exposure seem to be the result of homeostasis disruptions or responses that are dependent on stresses. Additionally, microplastics change and inhibit hormones linked to lipid metabolism, which eventually modifies the levels of blood cholesterol. A significant source of energy, cholesterol is also a necessary part of cell membranes, cell recognition mechanisms and signaling pathway. A considerable elevation in the serum cholesterol was observed in *Sparus aurata* after exposure to nanoplastics polymethylmethacrylate, which may cause long-term issues with energy balance or nutrition [12]. The liver controls the metabolism of lipids, including cholesterol, and microplastics may compromise the circulation of lipids. A noteworthy drop in the level of cholesterol and increase in total protein in *C. gariepinus* exposed with microplastic polyethylene. Exposure to polyethylene alters the level of blood cholesterol, suggesting that microplastic exposure can impact important structure of the cellular membrane and produce energy or nutritional related issues in fish. Following gill injuries, decreased osmotic regulation leading to plasma serum water loss maybe the cause of elevated serum total protein [52]. A substantial drop in *C. carpio* exposed to microplastics in both plasma total protein and cholesterol was observed [34]. While total proteins remain unaltered, polyvinylchloride dramatically raised the albumin level in serum of *S. aurata*, suggesting that osmotic equilibrium was unaffected [23]. The serum total protein levels of fish are altered by microplastic exposure, suggesting that microplastic exposure may impair cellular activity or protein synthesis. Numerous investigations have shown that exposure to microplastic alters the blood cholesterol, glucose and total protein content, indicating that microplastic exposure may cause alterations in lipid metabolism, glucose metabolism and osmotic disorders in fish [54].

Enzymatic plasma constituents, including ALP, ALT and AST, are regarded as sensitive and dependable biomarkers for evaluating the extent of tissue injury, including liver, when exposed to environmental stressors [54]. A considerable rise in ALP and ALT level of *C. carpio* after exposure to polyethylene, which may be brought on by the injury to the cellular membrane as well as mitochondria [8]. It was demonstrated that *O. niloticus* exposed to microplastics had considerably higher levels of ALP, ALT and AST, and this was linked to potential liver and cellular membrane injury [35]. On the other hand, Brandts et al. [13] reported that exposure with polymethylmethacrylate significantly reduced the levels of serum cholesterol, glucose, ALP, ALT and AST in *D. labrax*, maybe as a result of hemotoxicity affecting the blood constituents. It was found that a substantial rise in ALP level in polyethylene exposed *Symphysodon aequifasciatus*. Given that ALP is a key marker of liver injury, a considerable elevation of ALP level in *S. aequifasciatus* is thought to be the result from stress and liver injury brought on by microplastic exposure [98]. Because of injury and inflammation caused by the microplastic, which results in leakage of the enzymes from the hepatocytes. The notable increase was observed in the level of ALP and AST in *D. rerio* after exposured with polystyrene microplastics [91]. Exposure of microplastics polyvinylchloride (100–200 µm) at 10, 20 and 30 % concentrations for 60 days reduced the expression of ALP and acid phosphatase (ACP) in gut and liver of the larvae of *Cyprinus carpio*. In contrast, distinct outcomes were noted in the gills, where there was a notable rise in the activities of ALP and ACP. Though, as the concentration of the polyvinylchloride rose, the activities of ALP and ACP in the gill steadily diminished [60].

Fish exposed to protein-plastic complexes (13–600 nm size), microplastics enter into the bloodstream, they disturbing serum proteins that are necessary for immune response, osmotic pressure, lipid metabolism, molecular transportation and coagulation of the blood. Specifically, microplastic particles may have affected different physiology of the blood by executing the blood both physically as well as chemically. Hence, numerous investigations have verified changes to different hematological parameters after exposure to microplastic, and hematological parameters may be a useful markers to assess fish toxicity [54].

### 3.2 Neurotoxicity

Microplastic exposure can cause neurotoxicity in fish by impairing lipid peroxidation and causing disturb to the enzymes associated with the nervous system [54]. Numerous neurotransmitters, including melatonin, dopamine, oxytocin, vasopressin, aminobutyric acid, kisspeptin and serotonin, can be inhibited when microplastic is exposed to fish [11]. Acetylcholinesterase is one of the neurotransmitters that is most frequently employed as a primary signal of neurotoxicity because it can reveal probable neuromuscular cholinergic damage [10]. Acetylcholine, which is necessary for cholinergic neurotransmission at the cholinergic brain synapses and neuromuscular junctions, is inactivated by acetylcholine esterase, which is crucial for preserving healthy neuromuscular system function. Fish acetylcholine esterase activity is inhibited by microplastic, which negatively impacts cholinergic neurotransmission and may result in neuromuscular as well as neurological dysfunction [98]. The suppression of the acetylcholine esterase causes the acetylcholine level in brain to rise noticeably, causing the neurological system to dysfunction. Accumulation of the acetylcholine in the synaptic cleft impairs neurotransmission and overstimulates receptors, resulting in paralysis and death [17].

After being exposed to microplastic, level of the acetylcholine esterase in the brain of *D. labrax* diminishes, demonstrating that neurotoxicity brought on by the inhibition of acetylcholine esterase and harmful lipid peroxidation [10]. After exposure to microplastic, *O. niloticus* exhibits a decrease in the activity of acetylcholine esterase, which may result in intricate biochemical processes *e.g.* neurotoxicity [22]. Exposure of polyvinylchloride markedly reduced acetylcholine esterase level in the brain of *C. gariepinus*, leading to a build-up of acetylcholine at the synapses [47]. After exposure to polyethylene, *Pomatoschistus microps* showed acetylcholine esterase inhibition at a level high enough to have an adverse impacts on neurological activity ([67]). Motor dysfunction is brought on by neurotoxicity from microplastic exposure, which is thought to be the result of the suppression of the activity of acetylcholine esterase [16]. Numerous findings have revealed that microplastics exposure inhibits acetylcholine esterase in a variety of fish, which results in serious motor dysfunction, aberrant behavior as well as neurological disorders [16], [17], [10], [22], [8], [98], [46], [47], [104], [91], [95], [20]. Though, there were a few literature which supported that exposure of microplastics doesn’t affect the level of acetylcholine esterase in fish [25], [104], [80], [84]. Dietary exposure of polyvinylchloride microplastics (100 µm) at 0.5, 1.5 and 3.0 % in pellet for 45 days reduced the acetylcholine esterase level in brain at 0.5 % in pellet, but no change in the acetylcholine esterase level was observed in gills of *C. gariepinus* [47]. Similarly, exposure of microplastics polystyrene (size: 45 µm) at 1 mg/L for 120 hours did not affect the acetylcholine esterase level in the brain of *D. rerio*, but polystyrene nanoplastics (size: 50 nm) for the same duration and dose reduced brain acetylcholine esterase level in *D. rerio* [17]. Exposure of polystyrene microplastics (50 nm) at 0.5 mg/L for 1 and 2 days reduced the acetylcholine esterase level in the brain of *D. rerio*, whereas at day 3 there were no effect in the levels of acetylcholine esterase was reported [16]. Polystyrene microplastics (5 µm) at 10, 100 and 1000 µg/L for 1, 3 and 7 days did not impact the brain acetylcholine esterase level in the larvae of *Carassius auratus*. Polystyrene nanoplastics (70 nm) for the same dose and duration did not affect the level of acetylcholine esterase in the brain of *C. auratus* larva at day 1, but reduced the level at 1000 µg/L at day 3 as well as 100 and 1000 µg/L at day 7 [104]. Hence, many fish have been shown to experience neurological disturbances as a result of microplastic exposure, and acetylcholine esterase may be a sensitive and trustworthy biomarker for assessing microplastic induced toxicity [54] and its expression is dependent on dose, duration and size of the microplastics exposure.

### 3.3 Immune responses

The immune system of fish exposed to microplastic can be directly impacted by the damage of gut because of the production of cytokines as well as inflammation of the gut [11]. Fish immune systems can be impacted by microplastic exposure through the stimulation of oxidative burst activity, primary granule degranulation and neutrophil extracellular trap release. Accompanying this, microplastic that has been ingested by the fish may interact with the intestines and get into the bloodstream, impairing immune function modulation [41]. Fish are protected from foreign substances by their innate immunity, which balances the limitations of their adaptive immunity [54]. Fish innate immunity is triggered by the bioaccumulation of the microplastics [23]. Chemical toxicity and physical obstructions brought on by microplastic bioaccumulation in fish tissues may have an effect on the immunity by obstructing the uptake of nutrients and affecting the distribution of energy [35]. Plastic particles are the target of innate immune system of the cell, and positively charged microplastic nanoparticles bind favorably with negatively charged cellular membranes to facilitate its absorptivity through the cells [23]. Microplastics not only cause physical harm to the important immunological organs including the GIT and liver as well as to natural barriers of fish defense such as gills, but also enter into the cell, interfering with the intracellular signaling pathways, changes the cytokines level, upsetting the immunological homeostasis and eventually jeopardizing specific immunity [57].

Lysozyme plays a key role in fish innate immune system and can manifest itself in different ways based on the kind of toxins present as well as biological and environmental factors. The prevalent sort of WBCs in the circulation of fish are neutrophils. Fish are protected by differentiated leukocytes called neutrophils from numerous foreign contaminants, triggering potent antimicrobial responses in the early phases of invasion. In fish, phagocytosis is a significant immunological response, which is an old defense mechanism that involves intracellular translocation. Phagocytic cell granules contain peroxidase, and the release of this enzyme into the circulation is a crucial marker for assessing the immunologically effective state of flowing leukocytes in the circulation. Immunoglobulin in fish is a key antibody that keeps the immunity of fish working and is a vital immunological marker of adverse effects after exposure to several hazardous compounds [54]. Fish that are exposed to microplastics first attract a large numbers of T cells and macrophages while lysozymes are being activated. The immune cells eventually undergo apoptosis as a result of this exposure, a reduction in complement content, activity of lysozyme and lysosomal degradation capacity. Microplastics have a modest specific surface area and are effective at binding with viruses, organic contaminants and heavy metals to increase the immune response in the fish [57].

Exposure of microplastics polystyrene and polycarbonate enhanced the level of peroxidase and neutrophil in *Pimephales promelas*. In order to further encourage cell absorption, positively charged microplastics might fist interact with negatively charged cellular membranes. In fish, these two microplastics function as stressors, triggering innate immune responses [31]. When *S. aurata* was exposed to microplastic, which resulted in the decrease of phagocytosis and increase of immunoglobulin and peroxidase, however did not exhibit any notable alterations in the other organs (immunoglobulin did not exhibit any alterations in serum, and peroxidase did not exhibit any alterations in kidney and serum [23]. A notable decrease in the level of immunoglobulin and lysozyme after exposure with microplastic was observed in *C. carpio*. Inhibition of cholinesterase after exposure of microplastic in *C. carpio* may be the cause of the drop in the levels of immunoglobulin. In *C. carpio*, the reduction in the levels of lysozymes may inhibit the non-specific immune response [8]. After exposure to polyethylene, *D. labrax* developed higher levels of immunoglobulins, which affected innate immune system [24]. By obstructing nutrient absorption and affecting energy distribution, microplastic absorption decreased neutrophil numbers in *O. niloticus* [35]. Polystyrene (32–40 µm) exposure enhanced the expressivity of the cytokines associated with immunity such as IL-6, TLR4, IFN-γ and TNF-α in *Poecilia reticulata* [45]. Polyethylene microplastics at 100 and 1000 µg/mL for one week first enhanced and then reduced the levels of complement C3 and C4 of *Danio rerio* in a dose dependent mechanism and expression of the immune associated genes such IL-10, polymeric immunoglobulin receptor (PIGR) and immunoglobulin heavy variable4–5 (IGHV4–5) [111]. Exposure of microplastics polyvinylchloride (100–200 µm) at 10, 20 and 30 % concentrations for 60 days enhanced the generation of reactive oxygen species and upregulated the expression of pro-inflammatory cytokines such as TNF-α, IL-6 and IL-8 in the larvae of *Cyprinus carpio* [60]. Polystyrene (0.8–20 µm) exposure accumulated in the phagocytic B cells in *Oncorhynchus mykiss* [117]. Exposure of polystyrene microplastics (5 µm) at 50 and 500 µg/L for 21 days caused oxidative stress and inflammation in the intestine of the *D. rerio*. Significant changes were also seen in the metabolic profiles of the tissues and the intestinal microbiota; the majority of these changes were linked to oxidative stress, inflammation and metabolism of lipids [75]. Xu et al. [102] stated that microplastics triggered lipid peroxidation induces NFκB pathway and triggers ferroptosis, which in turn induce gut injury and apoptosis of the cells. Pro-inflammatory cytokines such as tumor necrosis factor-α (TNF-α) and interferon-γ (IFN-γ) are released when fish were exposed to microplastic particles, causing inflammation. Microplastics will induce the release of interleukin and TNF-α. *D. rerio* exposed to polystyrene plastics showed enhanced inflammation in the liver tissue and neutrophils counts; the effects were more pronounced when the plastic particle concentration was higher. Exposure of polyester microplastic particles to the *O. niloticus* caused increase level of TNF-α and IFN-γ. Concentration of the microplastics exposed to *O. niloticus* is directly correlated with the elevated concentration of the TNF-α and IFN-γ. When non-self substances like microplastic particles are encountered, T-helper cells and macrophages release the cytokine TNF-α and IFN-γ, which eventually activate the immune system [39]. When *P. reticulata* was exposed to 100 and 1000 µg/L microplastic particles for 28 days, the gut showed significant increase in the level of IL-6, TLR4 and IFN-γ [45]. The development of numerous diseases is linked to the overproduction of TNF-α. Overproduction of the inflammatory cytokines like TNF-α and IFN-γ can harm healthy cells. Both the inflammatory cytokines have the potential to induce severe inflammation [39]. As acknowledged foreign substances, microplastics have the ability to either stimulate or decrease the immune response by causing immunotoxicity *i.e.* microplastics can influence fish immune responses through a variety of mechanisms. Nevertheless, detailed studies on fish immunological functions after microplastic exposure are necessary [54]. Table 4 represents the immunological responses triggered by microplastics exposure.

**Table 4:** Immunological responses triggered by microplastics exposure in fish.

**Route of exposure**	**Applied organisms**	**Microplastics type**	**Microplastics size**	**Microplastics dose**	**Duration**	**Target organ**	**Response dose**	**Response**	**Reference**
**Immunoglobulin**									
**Seawater**									
**Dietary**	*Dicentrarchus labrax*	Polyethylene	40–150 µm	100 and 500 mg/kg	21 days	Plasma		→	[24]
						Skin mucus	500 mg/kg	↑	[24]
	*Sparus aurata*	Polyvinylchloride	40–150 µm	100 and 500 mg/kg	15 and 30 days	Serum		→	[23]
						Skin mucus	500 mg/kg	↑	
**Freshwater**									
**Waterborne**	*Cyprinus carpio*	Polyethylene	-	250 and 500 µg/L	30 days	serum	250 and 500 µg/L	↓	[8]
		Polypropylene	0.9085 ± 0.0312 mm	1 and 2.5 g/L	1–7 days	Serum		↑	[106]
**Dietary**	*Cyprinus carpio*	Polypropylene	0.9085 ± 0.0312 mm	100 and 250 mg/g	1–7 days	Serum		↑	[106]
**Lysozyme**									
**Saltwater**									
**Dietary**	*Sparus aurata*	Polystyrene	1–20 µm	25 and 250 mg/kg	21 days	Gut	Both	↑	[69]
**Freshwater**									
**Waterborne**	*Oreochromis niloticus*	Polystyrene	0.35 and 9 µm	250 and 500 µg/L	28 days	serum	0.35 µm	→	[3]
							9 µm	↑	
	*Cyprinus carpio*	Polyethylene	-	250 and 500 µg/L	30 days	serum	250 and 500 µg/L	↓	[8]
		Polyvinylchloride	100–200 µm	10, 20 and 30 %	60 days	Intestine and liver	10, 20 and 30 %	↑	[60]
	*Symphysodon aequifasciatus*	Polystyrene	32–40 µm	50 and 500 µg/L	30 days	Kidney		→	[97]
**Peroxidase**									
**Seawater**									
**Dietary**	*Dicentrarchus labrax*	Polyethylene	40–150 µm	100 and 500 mg/kg	21 days	Skin mucus	500 mg/kg	↓	[24]
	*Sparus aurata*	Polyvinylchloride	40–150 µm	100 and 500 mg/kg	15 and 30 days	Kidney		→	[23]
						Skin mucus	100 mg/kg	↑	
						Serum		→	
**Freshwater**									
**Waterborne**	*Pimephales promelas*	Polycarbonate	158.7 nm	0.025–0.2 µg/L	15 days	Kidney	0.025, 0.05, 0.1 and 0.2 µg/L	↑	[31]
**Neutrophil**									
**Freshwater**	*Oreochromis niloticus*	Microplastics	˃100 nm	1, 10 and 100 mg/L	15 days	Peripheral blood	1 mg/L	↓	[35]
	*Danio rerio*	Polyethylene + Polystyrene	41 nm	100 and 1000 µg/L	28 days	Gills, gut	Both	↑	[59]
	*Oncorhynchus mykiss*	Polystyrene	52.5 ± 11.5 µm	0.5, 2 and 5 %	42 days	-	5 %	↑	[42]
**Phagocytosis**									
**Seawater**									
**Dietary**	*Dicentrarchus labrax*	Polyethylene	40–150 µm	100 and 500 mg/kg	21 days	Kidney		→	[24]
						Skin mucus		→	
	*Sparus aurata*	Polyvinylchloride	40–150 µm	100 and 500 mg/kg	15, 30 days	Kidney		→	[23]
						Skin mucus	100 mg/kg	↑	
						serum		→	
					21 days	Skin mucus	500 mg/kg	↓	

↑: increase in response, →: no response, ↓: decrease in response

### 3.4 Oxidative stress

Micro- and nanoplastics produced toxicity by generating free radicals and can results in alterations in biochemistry, physiology, pathology and genomic instability, all are linked to the development of cancer. The precise pattern of oxidative stress brought on by microplastic exposure is difficult to pinpoint, although it has an impact on oxidative homeostasis. After exposure, microplastics bioaccumulated in the fish tissues, resulting in oxidative stress due to chemical and physical toxicity, endocrine disorders and blockage of the gut. Particularly, oxidative stress is the primary cause of microplastic toxicity in fish *viz.* disruption of the redox equilibrium, cellular damage and excessive formation of the reactive oxygen species (ROS) [54]. A discrepancy between the production of ROS and antioxidant defense of the fish is known as oxidative stress, and it can cause severe health problems, including cellular injury, DNA hydroxylation, lipid peroxidation, protein denaturation and apoptosis [43].

When the starting concentration of ROS rises quickly, toxicants cause fish to lose their redox equilibrium, rerouting energy to produce compounds and antioxidant enzymes that strengthen antioxidant activity. In order to counteract oxidative damage from ROS production, which is brought on by exposure to a variety of hazardous compounds, including the exposure of microplastic, fish have acquired antioxidant defense via modulating different antioxidant enzymes [54]. Actually, the antioxidant defense system of living organisms defends them against the damaging effects of naturally generating ROS through two different pathway: firstly, the enzymatic mechanism: with the help of antioxidant enzymes and secondly, a non-enzymatic pathway through the activities of glutathione, vitamin C, vitamin E and thioredoxin [43]. Superoxide dismutase (SOD) is the main defense mechanism against oxidative stress and it works by converting superoxide radical to hydrogen peroxide (H_2_O_2_). The enzyme catalase (CAT) breaks down H_2_O_2_ into H_2_O and O_2_. Glutathione S-transferase (GST) is participated in the transport of a variety amphiphilic and hydrophobic molecules such as steroids, heme and lipids as well as detoxification of numerous cytotoxic chemicals and provide defense against oxidative stress. Because GST linked with the phase II detoxification pathway and works a crucial part in inhibiting harm to various other biomolecules found in cells by facilitating the interaction of glutathione to foreign particles, including microplastics [54].

Antioxidant responses are triggered when bioaccumulation of the microplastic produces ROS in fish, which suggests a vital enhancement in the production of antioxidant enzymes [54]. *O. niloticus* showed enhanced activity of SOD after being exposed to polystyrene, which was explained as the antioxidant system creating ROS in response, leading to oxidative stress [22]. An elevation in the activities of CAT and SOD in *S. aequifasciatus* after exposure to polystyrene microplastic. An antioxidant response prevented oxidative injury brought on by the generation of ROS [97], [98]. *Poecilia reticulata* has enhanced the activities of CAT, SOD and GST upon exposure to microplastic polystyrene, owing to heightened generation of ROS [45]. Exposure of polyethylene microplastics (size: 22–71 µm) at ≥ 32 mg/L for 14 days triggered oxidative stress in gills, gut and liver of *Carassius carassius*, which eventually enhanced the levels of GST, CAT and SOD [110]. Microplastic markedly enhanced the GST level in *S. aurata*, suggesting that this is a detoxifying mechanism reducing the oxidative stress [86]. Polystyrene microplastics (size: 0.49–92 µm) exposure over concentration 5000 mg/L for 96 hours markedly enhanced the levels of GST, CAT and SOD in *Pseudobagrus fulvidraco* [55]. Exposure of polyamide microplastics markedly enhanced the activity of GST, CAT and SOD in the gut, gill and liver of *C. carassius* [18]. Exposure to microplastics polyethylene markedly reduced the activity of CAT, while enhanced the activity of SOD in *C. carpio* [9]. According to Romano et al. [79], *Carassius auratus* treated to microplastic polyvinylchloride showed a substantial rise in the activities of GST, suggesting that microplastics may shield cells against oxidative injury.

Because of the energy required to counteract oxidative stress, exposure of microplastic can also result in a reduction in antioxidant enzymes [36]. It has been shown that exposure to microplastics can prevent the typical ROS mediated oxidation pathway, resulting in a decrease in the activity of CAT in *Oryzias melastigma* [96]. Microplastic polystyrene exposure decreased the activity of CAT in the larvae of *D. rerio*, suggesting that the antioxidant defense becomes unbalanced due to oxidative injury [93]. According to Xia et al. [101], polyvinylchloride microplastic exposure reduced following an initial rise in SOD of the target tissue *i.e.* gills and gut of *Cyprinus carpio*. The inability of the antioxidant enzymes to quickly eliminate ROS generated in tissue was the reason given for this phenomenon. As microplastic concentration rose, activity of CAT similarly dropped, suggesting oxidative stress and injury. When *D. labrax* was exposed to polyethylene microplastics, the activities of CAT and SOD both dropped, possibly resulting in oxidative stress [24]. In order to combat oxidative stress, larvae of *C. auratus* exposed to low concentration of microplastics had higher activity of SOD, however, exposure to elevated levels of microplastics demolished the antioxidant defense, which resulted in decreased activity of SOD [104]. *O. melastigma* treated with microplastics polystyrene showed variable activity of GST depending on the type of tissue *viz.* reduced in the testis and liver, but enhanced in gills and gut [96]; conversely, *O. melastigma* showed varying GST activity in relation to size of the microplastic, with GST raising at 50 nm and falling at 45 µm [51].

Exposure of microplastics to the fish benefitted from glutathione (GSH) as it helps to maintain the redox state, while inhibit oxidative stress [97], [98]. Oxidized glutathione (GSSG) can be created when GSH and ROS are mixed together. Glutathione peroxidase (GPx) mediates the H_2_O_2_ breakdown pathway, which consumes GSSG [45]. An enzyme called GPx helps convert peroxide into less harmful hydroxyl molecules that shield cells from oxygen-induced injury. The reactant *i.e.* H_2_O_2_ first produces H_2_O and reduces GSH in order to oxidize GSSG (2GSH + H_2_O_2_ → GSSG + 2 H_2_0). Glutathione reductase (GR) then proceeds to decrease GSSG (GSSG + NADPH + H → 2GSH + NADP^+^) [86]. By reducing NADPH, the enzyme GR catalyzes the transformation of GSSG into a reduced GSH [80]. Consequently, the antioxidant response that eliminates free radicals produced by microplastic exposure is inherently linked to GSH as well as the enzymes that rely upon it. Hence, GSH may be used as a potent marker to clarify fish antioxidant defense after exposured to microplastics [54].

When *S. aequifasciatus* was exposed to microplastic polystyrene, GSH and GPx rose dramatically. After being exposed to microplastics, GPx may rise to prevent the production of ROS by neutralizing H_2_O_2_. A notable rise in the GSH level triggers the mechanism heavily dependent on glutathione for the antioxidant defense against oxidative stress [97], [98]. In *P. reticulata*, exposure to microplastic polystyrene resulted in a considerable decrease in the GSH level but enhanced the level of GR, GPx and GSSG. As concentration of the microplastic rises, GSH reduces because it interacts with the ROS, which raises GSSG. Activity of GR then rises, possibly as a result of microplastic influenced ROS generation, increasing levels of GSSG to GSH [45]. Additionally, Wan et al. [93] found that the larvae of *D. rerio* exposured with microplastic polystyrene had a significantly lower GSH level. A notable drop in the activity of GPx in *C. carpio* after exposure to polyvinylchloride microplastic, potentially as a result of antioxidant function dropping when stress coming from ROS surpassed the threshold [101]. A noteworthy reduction in the GSH level in the larvae of *D. rerio* subjected to microplastic polystyrene exposure. However, exposure of microplastic did not change the activity of GPx, suggesting that the redox buffer of the GSH has been depleted [16], [17]. Exposure of polyethylene microplastics (size: 22–71 µm) at ≥ 32 mg/L for 14 days triggered oxidative stress in gills, gut and liver of *Carassius carassius*, which eventually reduced the level of GSH [110]. Exposure to microplastics polyethylene markedly reduced the activity of GR, while enhanced the activity of GPx in *C. carpio* [9]. Exposure of polyamide microplastics markedly enhanced the activity of GSH in the gut, gill and liver of *C. carassius* [18]. Exposure of polystyrene microplastics (size: 0.49–92 µm) over concentration 5000 mg/L for 96 hours markedly reduced the levels of GSH in *P. fulvidraco* [55]. Exposure to microplastic polystyrene reduced the activity of GPx in *D. rerio*, inhibiting H_2_O_2_ from turning into a harmless OH^-^ substrate, which would cause bioaccumulation of ROS in tissues [91].

Since malondialdehyde (MDA) is the end product of oxidative injury to the lipids, and utilized as bio-indicator of lipid peroxidation [4]. A discrepancy between the production of ROS and antioxidant defense of the fish is known as oxidative stress, and it can cause severe health issues, including cellular injury, lipid peroxidation, DNA hydroxylation, apoptosis as well as protein denaturation [43]. A significant indicator of toxic responses is an increase in ROS, which is frequently generated by mitochondria and the generation of ROS is initiated by injury to the mitochondria. When the larvae of *C. auratus* were exposed to microplastic, their levels of ROS increased, and the size of the microplastic has an effect as well on ROS [104]. When *D. rerio* was exposed to microplastics polystyrene, lipid peroxidation and ROS dramatically increased. Elevated amounts of ROS cause lipid peroxidation, which harm the structural integrity of the cellular membrane by generating MDA and 4-hydroxynonenal (Umamaheswari et al., 2020).

Lipid peroxidation is a series of events brought on by free radicals, including oxygen radicals. Increased lipid peroxidation in *D. labrax* treated with microplastics (fluorescence red polymer microspheres) suggests that lipid damage and oxidative stress are brought on [10]. After exposure to microplastic, lipid peroxidation in *O. niloticus* increased considerably. As a byproduct of lipid peroxidation, MDA serves as a biomarker for oxidative stress brought on by exposure to different hazardous chemicals, which damages tissues [36]. When *O. niloticus* was exposed with microplastics polystyrene, MDA level was dramatically increased, perhaps as a result of oxidative stress [22]. After treating with microplastic polystyrene, MDA level dramatically enhanced in *O. melastigma*, which was associated with the ROS generation and oxidative damage of lipids [96]. After exposure with microplastic polystyrene, MDA level dramatically enhanced in *S. aequifasciatus*, with enhanced MDA level perhaps causing oxidative stress as well as lipid peroxidation [97]. On contrary, MDA level was found to be reduced in *C. carpio* after exposure to polyvinylchloride microplastic [101].

Even in cases when the detoxification systems and antioxidant response such as GSH, GST, CAT and SOD were engaged, exposure of microplastic to *S. aurata* resulted in enhanced oxidative stress, lipid peroxidation and protein denaturation [86]. Because of the huge surface area, microplastic have the potential to cause oxidative stress via the production of ROS during an inflammatory response or oxidizing molecules that have been absorbed on the surface [71]. Fish exposed to microplastics provoked generation of ROS, which either inhibits or stimulates antioxidant responses. Furthermore, microplastic exposure in fish disrupts or affects the glutathione and its associated responses in fish, which support to antioxidant responses. Exposure of microplastics results in an excess ROS production in the mitochondria of the fish hepatocytes. ROS stimulates toll-like receptor 2 (TLR2) expression and triggers the downstream molecules myeloid differentiation factor 88 (MyD88), tumor necrosis factor receptor-associated factor 6 (TRAF6) of the TLR2 signaling pathway, eventually contributing NFκB p65 phosphorylation. This causes oxidative stress, inflammation, and the production of inflammatory factors into the liver of fish [88]. Hence, fish exposed to different microplastics experience oxidative damage induced by oxidative stress as a result of the disruption of the antioxidant homeostasis between generation of ROS and antioxidant defense [54]. The antioxidant parameters associated with oxidative stress generated by microplastic exposure in fish are depicted in Table 5.

**Table 5:** The antioxidant indices linked to oxidative stress in fish produced after the exposure of microplastics.

**Route of exposure**	**Applied organism**	**Microplastics type**	**Microplastics size**	**Microplastics dose**	**Duration**	**Target organs**	**Response dose**	**Response**	**Reference**
**Catalase**									
**Saltwater**									
**Dietary**	*Dicentrarchus labrax*	Polyethylene	40–150 µm	100 and 500 mg/kg	21 days	Liver	100 and 500 mg/kg	↓	[24]
		Polyvinylchloride	40–150 µm	100 and 500 mg/kg	21 days	Liver	100 and 500 mg/kg	→	
	*Sparus aurata*	Low density polyethylene	200–500 µm	0.5–10 % of feed	30, 60, 90 and 120 days	Intestine		↑	[86]
**Waterborne**	*Oryzias melastigma*	Fluorescent polystyrene	50 nm	10 µg/mL	14 days	Liver and intestine	10 µg/mL	↑	[51]
			45 µm					↓	
		Polystyrene	10 µm	2, 20 and 200 µg/L	60 days	Testis	2, 20 and 200 µg/L	↓	[96]
						Liver	20 and 200 µg/L	↓	
						Gut	200 µg/L	↓	
**Freshwater**									
**Waterborne**	*Carassius auratus* larvae	Polystyrene	70 nm	10, 100 and 1000 µg/L	1 days	-	100 and 1000 µg/L	↓	[104]
					3 days	-	1000 µg/L	↓	
					7 days	-	100 and 1000 µg/L	↓	
			50 µm	10, 100 and 1000 µg/L	1 days	-	100 and 1000 µg/L	↓	
					3 days	-	1000 µg/L	↓	
					7 days	-		→	
	*Carassius auratus*	Polyvinylchloride	0.1–1000 µm	0.1 and 0.5 mg/L	96 hours	Brain, liver	0.5 mg/L	↑	[79]
	*Cyprinus carpio*	Polyvinylchloride	100–200 µm	10, 20 and 30 % of weight	30 and 60 days	30 days	60 days		
					Liver	-	20 and 30 %	↓	[101]
					Gut	10, 20 and 30 %	20 and 30 %	↓	
					Gill	10, 20 and 30 %	20 and 30 %	↓	
		Polypropylene	0.9085 ± 0.0312 mm	1 and 2.5 g/L	1–7 days		↓	[106]	
	*Poecilia reticultata*	Green fluorescent polystyrene	32–40 µm	100 and 1000 µg/L	28 days	Visceral	1000 µg/L	↑	[45]
	*Symphysodon aequifasciatus*	Polystyrene	32–40 µm	50 and 500 µg/L	30 days	Liver	Both	↑	[97]
	*Danio rerio* larvae	Red fluorescent microplastics	1–5 µm	2 mg/L	96 hours post fertilization	-	-	↑	[80]
		Pristine polystyrene	5 and 50 µm	100 and 1000 µg/L	7 days	-	1000 µg/L	↓	[93]
	*Danio rerio*	Fluorescent polystyrene	70 nm	20, 200 and 2000 µg/L	4 and 12 hours, 1, 2 and 7 days	Liver	2000 µg/L	↑	[61]
			5 µm				200 and 2000 µg/L	↑	
	*Oreochromis niloticus*	Microplastics	˃100 nm	1, 10 and 100 mg/L	15 days	Serum	10 and 100 mg/L	↑	[36]
	*Paramisgurnus dabryanus* juveniles	Polystyrene	0.5 and 5 µm	100 or 1000 µg/L	21 days			↓	[95]
**Dieatry**	*Cyprinus carpio*	Polypropylene	0.9085 ± 0.0312 mm	100 and 250 mg/g	1–7 days			↓	[106]
**Superoxide dismutase**									
**Saltwater**									
**Dietary**	*Dicentrarchus labrax*	Polyethylene	40–150 µm	100 and 500 mg/kg	21 days	Liver	500 mg/kg	↓	[24]
		Polyvinylchloride						→	
	*Sparus aurata*	Low density polyethylene	200–500 µm	0.5–10 % of feed	30, 60, 90 and 120 days	Intestine		↑	[86]
**Waterborne**	*Oryzias melastigma*	Fluorescent polystyrene	50 nm	10 µg/mL	14 days	Liver, Intestine	10 µg/mL	↑	[51]
			45 µm			Intestine		↓	
		Polystyrene	10 µm	2, 20 and 200 µg/L	60 days	Testis	200 µg/L	↓	[96]
						Gill	20 and 200 µg/L	↑	
						Liver	200 µg/L	↓	
						Gut	2 and 20 µg/L	↑	
**Freshwater**									
**Waterborne**	*Carassius auratus* larvae	Polystyrene	70 nm	10, 100 and 1000 µg/L	1 days			→	[104]
					3 days		10 µg/L	↑	
					7 days		1000 µg/L	↓	
			50 µm		1 days		1000 µg/L	↑	
					3 days			→	
					7 days		100 µg/L	↑	
	*Carassius auratus*	Polyvinylchloride	0.1–1000 µm	0.1 and 0.5 mg/L	96 hours	Liver	0.5 mg/L	↑	[79]
	*Cyprinus carpio* larvae	Polyvinylchloride	100–200 µm	10, 20 and 30 % of weight	30 and 60 days	30 days	60 days		
					Gill	20 and 30 %	10, 20 and 30 %	↓	[101]
					Gut	20 and 30 %	10, 20 and 30 %	↓	
					Liver	10, 20 and 30 %	10, 20 and 30 %	↓	
	*Poecilia reticulata*	Green fluorescent polystyrene	32–40 µm	100 and 1000 µg/L	28 days	Visceral	Both	↑	[45]
	*Symphysodon aequifasciatus*	Polystyrene	32–40 µm	50 and 500 µg/L	30 days	Liver	500 µg/L	↑	[97]
	*Paramisgurnus dabryanus* juveniles	Polystyrene	0.5 and 5 µm	100 or 1000 µg/L	21 days			↓	[95]
	*Oreochromis niloticus*	Green fluorescent polystyrene	0.1 µm	1, 10 and 100 µg/L	1, 3, 6, 10 and 14 days	Liver	1, 10 and 100 µg/L	↑	[22]
		Polystyrene	1 µm	0.01, 0.1 and 1 mg/L	14 days			↑	[20]
		Microplastics	˃100 nm	1, 10 and 100 mg/L	15 days	Serum	1, 10 and 100 mg/L	↑	[36]
	*Danio rerio* larvae	Pristine polystyrene	5 and 50 µm	100 and 1000 µg/L	7 days			→	[93]
	*Danio rerio*	Polystyrene	0.1–0.12 µm	10 and 100 µg/L	7, 14, 21, 28 and 35 days	Liver, brain	Both	↓	[91]
			150 µm	75 mg/L	12 and 24 days	Liver, gills	75 mg/L	↓	[103]
		Pristine polystyrene	0.1 µm	200 µg/L	14 days	Liver, Intestine	200 µg/L	↓	[74]
			20 µm					→	
		Fluorescent polystyrene	70 nm	20, 200 and 2000 µg/L	4 and 12 hours, 1, 2 and 7 days	Liver	2000 µg/L	↑	[61]
			5 µm				20, 200 and 2000 µg/L	↑	
**Glutathione S-transferase**									
**Saltwater**									
**Waterborne**	*Oryzias melastigma*	Fluorescent polystyrene	50 nm	10 µg/mL	14 days	Liver, Intestine	10 µg/mL	↑	[51]
			45 µm					↓	
		Polystyrene	10 µm	2, 20 and 200 µg/L	60 days	Testis	20 and 200 µg/L	↓	[96]
						Gill		↑	
						Liver		↓	
						Gut		↑	
**Freshwater**									
**Waterborne**	*Carassius auratus*	Polyvinylchloride	0.1–1000 µm	0.1 and 0.5 mg/L	96 hours	Gill, brain, liver	0.5 mg/L	↑	[79]
	*Poecilia reticulata*	Green fluorescent polystyrene	32–40 µm	100 and 1000 µg/L	28 days	Visceral	Both	↑	[45]
	*Danio rerio* larvae	Red fluorescent microplastics	1–5 µm	2 mg/L	96 hours post fertilization			→	[80]
	*Danio rerio*	Polystyrene	0.1–0.12 µm	10 and 100 µg/L	7, 14, 21, 28 and 35 days	Liver, brain	Both	↑	[91]
			150 µm	75 mg/L	12 and 24 days			→	[103]
**Glutathione**									
**Saltwater**									
**Dietary**	*Sparus aurata*	Low density polyethylene	200–500 µm	0.5–10 % of feed	30, 60, 90 and 120 days	Intestine		↑	[86]
**Waterborne**	*Oryzias melastigma*	Polystyrene	10 µm	2, 20 and 200 µg/L	60 days	Testis	20 and 200 µg/L	↓	[96]
						Gill	200 µg/L	↑	
						Liver	2, 20 and 200 µg/L	↓	
						Gut	200 µg/L	↑	
**Freshwater**									
**Waterborne**	*Danio rerio* larvae	Red fluorescent microplastics	1–5 µm	2 mg/L	96 hours post fertilization			→	[80]
		Polystyrene	50 nm and 45 µm	1 mg/L	5 days		1 mg/L	↓	[17]
		Pristine polystyrene	5 and 50 µm	100 and 1000 µg/L	7 days		Both	↓	[93]
	*Symphysodon aequifasciatus*	Polystyrene	32–40 µm	50 and 500 µg/L	30 days	Liver	500 µg/L	↑	[97]
	*Poecilia reticulata*	Green fluorescent polystyrene	32–40 µm	100 and 1000 µg/L	28 days	Visceral	Both	↓	[45]
	*Cyprinus carpio*	Polypropylene	0.9085 ± 0.0312 mm	1 and 2.5 g/L	1–7 days			↑	[106]
**Dietary**	*Cyprinus carpio*	Polypropylene	0.9085 ± 0.0312 mm	100 and 250 mg/g	1–7 days			↑	[106]
**Glutathione peroxidase**									
**Saltwater**									
**Dietary**	*Sparus aurata*	Low density polyethylene	200–500 µm	0.5–10 % 0 f feed	30, 60, 90 and 120 days	Intestine		↑	[86]
**Waterborne**	*Oryzias melastigma*	Polystyrene	10 µm	2, 20 and 200 µg/L	60 days	Testis	20 and 200 µg/L	↓	[96]
						Gill	200 µg/L	↑	
						Liver	2, 20 and 200 µg/L	↓	
						Gut	200 µg/L	↑	
**Freshwater**									
**Waterborne**	*Poecilia reticulata*	Green fluorescent polystyrene	32–40 µm	100 and 1000 µg/L	28 days	Visceral	Both	↑	[45]
	*Symphysodon aequifasciatus*	Polystyrene	32–40 µm	50 and 500 µg/L	30 days	Liver	Both	↑	[97]
	*Paramisgurnus dabryanus* juveniles	Polystyrene	0.5 and 5 µm	100 or 1000 µg/L	21 days			↓	[95]
	*Carassius auratus*	Polyvinylchloride	0.1–1000 µm	0.1 and 0.5 mg/L	96 hours			→	[79]
	*Carassius auratus* larvae	Polystyrene	70 nm	10, 100 and 1000 µg/L	1 day		10, 100 and 1000 µg/L	↑	[104]
					3 days		10 µg/L	↑	
					7 days		1000 µg/L	↓	
			50 µm		1 day			→	
					3 days		1000 µg/L	↓	
					7 days			→	
	*Danio rerio*	Polystyrene	0.1–0.12 µm	10 and 100 µg/L	7, 14, 21, 28 and 35 days	Liver, brain	Both	↓	[91]
	*Cyprinus carpio*	Polyvinylchloride	100–200 µm	10, 20 and 30 % of weight	30 and 60 days	30 days	60 days		[101]
					Gill		10, 20 and 30 %	↓	
					Gut			↓	
					Liver			↓	
**Glutathione reductase**									
**Saltwater**									
**Dietary**	*Dicentrarchus labrax*	Polyvinylchloride	40–150 µm	100 and 500 mg/kg	21 days			→	[24]
		Polyethylene						→	
**Freshwater**									
**Waterborne**	*Carassius auratus*	Polyvinylchloride	0.1–1000 µm	0.1 and 0.5 mg/L	96 hours			→	[79]
	*Poecilia reticulata*	Green fluorescent polystyrene	32–40 µm	100 and 1000 µg/L	28 days	Visceral	Both	↑	[45]
**Oxidized glutathione**									
**Freshwater**									
**Waterborne**	*Danio rerio* larvae	Red fluorescent microplastics	1–5 µm	2 mg/L	96 hours post fertilization			→	[80]
	*Poecilia reticulata*	Greeen fluorescent polystyrene	32–40 µm	100 and 1000 µg/L	28 days	Visceral	Both	↑	[45]
**Malondialdehyde**									
**Saltwater**									
**Dietary**	*Sparus aurata*	Low density polyethylene	200–500 µm	0.5–10 % of feed	30, 60, 90 and 120 days	Intestine		↑	[86]
**Waterborne**	*Oryzias melastigma*	Polystyrene	10 µm	2, 20 and 200 µg/L	60 days	Testis, gill, liver and gut	2, 20 and 200 µg/L	↑	[96]
**Freshwater**									
**Waterborne**	*Poecilia reticulata*	Green fluorescent polystyrene	32–40 µm	100 and 1000 µg/L	28 days	Visceral	Both	↑	[45]
	*Oreochromis niloticus*	Green fluorescence polystyrene	0.1 µm	1, 10 and 100 µg/L	1, 3, 6, 10 and 14 days	Liver		→	[22]
		Polystyrene	1 µm	0.01, 0.1 and 1 mg/L	14 days			↑	[20]
	*Symphysodon aequifasciatus*	Polystyrene	32–40 µm	50 and 500 µg/L	30 days	Liver	500 µg/L	↑	[97]
	*Danio rerio*	Pristine polystyrene	0.1 µm	200 µg/L	14 days	Liver	200 µg/L	↑	[74]
			20 µm			Liver		↑	
						Intestine		↓	
	*Cyprinus carpio*	Polypropylene	0.9085 ± 0.0312 mm	1 and 2.5 g/L	1–7 days			↑	[106]
		Polyethylene	-	175, 350, 700 and 1400 µg/L	30 days			↑	[9]
**Dietary**	*Cyprinus carpio*	Polypropylene	0.9085 ± 0.0312 mm	100 and 250 mg/g	1–7 days			↑	[106]

↑: increase in response, →: no response, ↓: decrease in response

### 3.5 Growth inhibition

Microplastics are a novel class of environmental contaminant that have generated significant interest due to their biological toxicity. There is microplastic pollution in the rivers, soil and oceans. The health and safety of aquatic organisms have been gravely jeopardized. The intestine serves as the primary organ for digestion, absorption as well as immunity and is essential to growth and proper maintenance of the metabolism. The primary role of the floral composition, a microbial ecosystem that housed in the gut, is to regulate the different physiological processes of the host species, including production of amino acids as well as proteins and encouraging the absorption of minerals. Several investigations have exhibited that microplastics upset the equilibrium of gut flora, which resulted in the enhanced richness of the harmful pathogens, while reduced the beneficial ones. Simultaneously, the reduced mucus layer and the structure of the gut villi point to a compromised barrier function of the gut [108]. Exposure of microplastic polystyrene (32–40 µm) at 100 and 1000 µg/L dose for 28 days was done in *Poecilia reticulata*. Authors found that microplastics dwell in the intestine and cause the goblet cells to expand in size. Authors recommended that microplastics may influence on the microbial role and composition in gut, including repair and metabolism associated pathway. Microplastic exposure trigger microbial dysbiosis to happen in the gut, which eventually reduced the nutrient utilization and overall growth performance of *P. reticulata* [45]. Polystyrene microplastics (5 µm) exposure at 1000 µg/L for 21 days caused a change in the microbial composition of the gut and metabolic disorders associated with the metabolism of energy in *Oryzias javanicus* [92]. Following exposure to polyethylene microplastics, the microbial composition of the gut were significantly altered and several pathogenic bacteria were discovered there as well in *C. carassius* [44]. Exposure of polystyrene nanoplastics (100 nm) at 10 mg/L for 28 days impact the immune response and microbial composition of the gut, directed to microbial dysbiosis in the gut of *Monopterus albus* [115]. Hence, microplastic exposure affect the intestinal health, microbial composition and thus creating obstacles in the digestion, absorption as well as metabolism of nutrients, hence affecting the overall growth performance of the fish.

The bioaccumulation of microplastics can lead to satiety and impair intestinal digestive performance, which eventually impair growth and decline survivability. Microplastic may also impede the actions of the fish digestive enzymes. Exposure of microplastic polystyrene (32–40 µm) at 100 and 1000 µg/L dose for 28 days lowered the digestive enzymes activity, including lipase, amylase, chymotrypsin and trypsin in *P. reticulata* [45]. Polyvinylchloride microplastic exposure impede the growth, body length and weight gain in the larvae of *C. carpio* [101]. The growth of the fish exposed to microplastics was not affected; however, the excretion of microplastics caused a significant reduction in growth, suggesting that the effect on growth occurred later in *C. carpio* [68]. Zhang et al. [113] proposed that, exposure of microplastics polyamide (32.50 µm) showed increased obstruction on weight and body length of the developing *D. rerio*. *D. rerio* found it more challenging to purify the microplastics in the gut, resulting in the loss of gut folding, gut microvilli emaciation and increased enterocytes shedding. Polyamide hindered the breakdown of dietary lipids in the larvae by causing lipid peroxidation, which in turn decreased the activity of lipase and the release of bile acids. Microplastics exposure downregulated the expression of genes linked to resynthesis as well as transportation of triglyceride *viz.* microsomal triglyceride transfer protein (MTTP), diacylglycerol O-acyltransferase 1 (DGAT1), diacylglycerol O-acyltransferase 2 (DGAT2), and cluster of differentiation (CD36), leading to maladsorption of lipid and suppression of growth. Mbugani et al. [65] exhibited that, exposure of polyethylene microplastics decreased growth performance in the juveniles of *Oreochromis urolepis* in terms of specific growth rate (SGR), total length, weight gain and final weight. They also observed the histomorphological lesion index (HLI) of the small intestine and argued that rise in HLI is correlated with the dosage of the polyethylene microplastic exposure. HLI of the small intestine did not significantly correlated with SGR, but it did with condition factors, growth pattern, total length, weight gain and final weight. As HLI enhanced, the allometric pattern of growth shifted towards an isometric one. These results imply that microplastics caused structural damage to the small intestine, therefore impairing the process involved in digestion as well as absorption of the nutrients, which eventually hampered the growth of *O. urolepis*. Exposure of polystyrene microplastics (5 µm spherical) has a major impact on gut microbial community structure and abundance, which may have an impact on the metabolic role of the GIT in the *Trachinotus blochii*. According to the metabolomics sequencing of the gut constituents, microplastics interfered with the metabolism of amino acid, glucose and lipids, hence jeopardizing the regular process of digestion as well as absorption of the gut. Furthermore, the stimulation of many pathways, such as signal transduction, metabolism of proline and endocrine system of gut, might result in the development of multiple disorders [105]. In short, microplastics cause intestinal injury, harm to the intestinal microbial composition, reduced the activity of the digestive enzymes and metabolism, thus affecting overall growth of the fish.

Dietary exposure of the microplastics polyethylene (27 and 63 µm) reduced weight gain, while enhanced hepatosomatic index and feed conversion ratio (FCR) in genetically improved farmed tilapia (GIFT), *O. niloticus*. Polyethylene microplastics also lowered the amylase and protease activity in gut. The altered microbial composition of the gut after microplastic exposure are associated with metabolism of nutrients and eventually affecting growth performance. Polyethylene microplastics having greater size (63 µm) were primarily found in the fecal matter, however, the smaller ones (27 µm), tended to bioaccumulate in the gut, hence size of the microplastic has greater influence on the growth performance of fish [62]. Zhang et al. [112] reported that both the microbial composition and diversity of gut are affected in large particle size (200 µm) compared to small particle size (2 and 10 µm) of polystyrene microplastics at 10 mg/L for 60 days in *O. melastigma*. The lipid content in liver, size of the adipocytes and body weight were considerably enhanced when fish are exposed to large (200 µm) polystyrene microplastic particles. The elevated body weight was associated with reduced the richness of Fusobacteria and increased the richness of Firmicutes/Bacteriodetes ratio and Verrucomicrobia. They have concluded that when fish are exposed to large sized polystyrene microplastics, their gut microbiota ought to be a major factor in controlling the lipid metabolism of the host species. It was reported that polyethylene microplastics (1–4 µm) at 1000 µg/L for 7 days affect the metabolism and microbial composition of the larvae of *D. rerio* [114]. Hence, size of the microplastic particles has a definite role to play in the growth performance of the fish.

Exposure of microplastic polyvinylchloride at 0.5 mg/L for 4 days decreased the expression of growth hormone receptor (GHR) and insulin like growth factor binding protein 1 (IGFBP1) in liver, while the expression of the cortisol receptor was enhanced. The liver is the primary site of production for insulin like growth factor 1 (IGF 1). IGF 1 acts as a key influencer of the growth inducing activity of growth hormone (GH), which directly promotes tissue growth. The efficacy of GH on target organs, mainly liver, are brought on via interactions with the membrane bound GHR. The transcription of the targeted IGF 1 gene is stimulated when GH binds to GHR and triggers a receptor mediated signaling cascade. It is therefore likely that the ability of GH to interact with GHR is diminished, as evidenced by the decreased expressivity of GHR in liver tissue in the present study. A decreased transcript of GHR may reduce the production of IGF 1, which could eventually result in the growth cessation. Additionally, IGFBP also controls the expression and biological activity of IGF 1. IGFBP is produced in several organs, released into the bloodstream or extracellular fluid to generate a binary complex along with IGF 1. These are suggested to lengthen the half-life of IGF 1 and facilitate its transportation to the intended location while maintaining its biological activity. The biological half-life would be shortened and eventually its growth inducing role would be diminished by a lower quantity of IGFBP 1 protein secreted into the bloodstream. Hence, the growth regulating genes that regulate the IGF 1/GH axis were negatively affected by the exposure of polyvinylchloride microplastics (0.5 mg/L) [79], eventually affecting the growth of the fish.

Microplastics can accumulate in the liver and caused toxicity. Liver is an important organ associated with metabolism, hence the growth of the infected organism also gets affected. Polyethylene microplastics at low and median doses (6.38 and 12.18 mg/fish/day) for 30 days induced the growth of *C. carassius*, while high dose at 22.33 mg/fish/day markedly reduced the growth of *C. carassius*. Groups that are treated with polyethylene microplastics showed signs of significant damage of liver tissue. In contrast to the control group, the medium and high polyethylene groups exhibited disordered hepatic tissue and necrosis of the acinar epithelial cells of the pancreas [44]. Hao et al. [37] conducted a survey on the exposure of polystyrene microplastics of various sizes (0.5 and 15 µm) and concentrations (100 and 500 µg/L) for 7 and 14 days in *Ctenopharyngodon idella*. They observed that, *C. idella* showed notable size as well as concentration dependent responses when exposed to microplastic toxicity. Growth rate of the fish clearly reduced on day 14 after being exposed to microplastic, but definitely not on day 7. Furthermore, large-sized and highly concentrated microplastics resulted in lower weight gain and more potential injury caused to liver, conversely, small-sized and highly concentrated microplastics caused higher oxidative stress and more severe hepatic congestion. Zhang et al. [112] conducted a study on exposure of polystyrene microplastics (size: 2, 10 and 200 µm) at 10 mg/L for 60 days to observe its effect in *O. melastigma*. The fish exposed to polystyrene microplastics showed signs of liver toxicity and lipid metabolic disorders, with particle size being the main contributing factor. When fish exposed to 200 µm particle size, the lipid content in liver, size of the adipocytes and body weight considerably enhanced, while fish treated with 2 and 10 µm particles exhibited signs of liver damage, primarily in the form of little inflammation and fibrosis. They have argued that this probably happening because large particles cannot enter into the bloodstream. Exposure of polystyrene nanoplastics (100 nm) at 10 mg/L for 28 days markedly upregulated the expression of Bax, Capase 9, Hsp70 and TGF-β in *Monopterus albus*. It is obvious that nanoplastics induce immune response as well as apoptosis in hepatic tissues [115]. Hence, microplastics exposure damage the liver tissue, which is a pivotal organ associated with the metabolism in a size and dose dependent mechanism, thus affecting the growth of the fish.

Food security for humans is greatly dependent on the quality as well as quantity of the aquaculture production. Aquacultural production and human health are at serious risk due to the introduction of new contaminants including microplastics and antibiotics into the aquatic environment. Antibiotics are extensively employed as therapeutic medications in animals, aquaculture and human because of their affordability, simplicity of application and effectiveness. Currently, it is estimated that over 1 lakh tonnes of antibiotics consumed annually worldwide. The misuse of antibiotics has changed the growth performance of the cultured animals, which has an impact on yield and quality of the aquacultural products. Furthermore, microplastics can absorb antibiotics by hydrophobic, electrostatic and distributive interaction due to their vast surface area and hydrophobicity, modifying their bioavailability. Exposure of microplastics along with antibiotics markedly decreased the SGR and weight gain rate in the juveniles of *Sebastes schlegelii*. It was revealed that there was a synergistic effects of the combined exposure on the suppression of growth. Hence, there is a potential risk of combined exposure of microplastics and antibiotics in the culture environment [90]. Li et al. [58] conducted a survey in *Cyprinus carpio* by exposed them with microplastics polyvinylchloride along with two antibiotics *i.e.* sulfamethazine or oxytetracycline for 8 weeks. Exposure of microplastics increased the diversity of the pathogenic bacteria in the gut of *C. carpio*. Surprisingly, polyvinylchloride microplastics have a greater impact on the diffusion and dissemination of antibiotic resistance genes than do antibiotics, which could result to antibiotic resistance in aquaculture. Hence, it is essential to carry out more scientific research to know the exact mechanism of the synergistic role played between microplastics and antibiotics in aquatic ecosystem and their effect on fish health.

### 3.6 Reproductive disruption

The aquatic biota is increasingly at risk from microplastic contamination, which may impair their physiological functions. However, little is known about how microplastics could exert the reproductive toxicity in fish [99]. The hypothalamic-pituitary-gonadal (HPG) axis, which regulates the interaction of three main organs *i.e.* hypothalamus, pituitary and gonad, that controls the reproductive processes of the majority of fish same as other vertebrates ([63]). The hypothalamus responds to both internal and external stimuli by generating gonadotropin-releasing hormone (GnRH). Neurons in teleosts fish innervate the pituitary gland more or less directly by projections to the vicinity of the pituitary gonadotrophs. Dopamine and GnRH are some of the neuropeptides and neurotransmitters that are released by these nerve endings. Gonadotrophin (GTH), a glycoprotein hormone, stimulates gonadal development as well as maturation in most vertebrates. Many teleosts, have been found to contain two types of GTHs *viz.* GTH-I, which is comparable to follicle stimulating hormone (FSH) and GTH-II, which is comparable to luteinizing hormone (LH), have been reported, which, while being found in many cells, work together to promote the synthesis of 17β-estradiol and in turn, the synthesis of sex steroids. However, only GTH-II has been found in primitive teleosts fish, such as eels and catfish, and it is recognized to regulate the entire development process of the gonad. The interaction between dopamine and GnRH function as inhibitory and stimulatory on the synthesis of LH and to a lesser extent FSH in many fish, but not restricted to all of them. The precise neuronal activation that triggers puberty and sexual maturation ultimately relies on a complicated interplay between external as well as internal stimuli. These includes growth factor, sex steroids and a number of other peripheral hormones; nevertheless, little is known about their targets. To regulate early steroidogenesis, gametogenesis, ovulation as well as spermiation, the anterior portion of the pituitary produces LH and FSH in response to GnRHs derived from the hypothalamus. Puberty is therefore regulated by GnRH and some other gonadal sex steroids. The release of the GnRH is controlled by a variety of neuropeptides and neurotransmitters [85].

The production and release of sex hormones are regulated by the HPG axis, and sirtuin 1 (SIRT1) is essential to this process. Exposure of polystyrene microplastics (50 and 500 µg/L) markedly altered gonadosomatic index (GSI) and reduced fecundity rate in *D. rerio*. An imbalance in hormonal homeostasis was revealed by the following alteration in serum sex hormone contents (17β-estradiol/testosterone). The changed gene transcription levels suggested that polystyrene microplastics may be interfering with the ability of the HPG axis to act properly. A study using molecular docking demonstrates that microplastics effectively interact to and inhibit SIRT1 and endocrine receptors [33]. Sayed et al. [82] reported that Microplastics ingestion markedly reduced the levels of FSH, LH, sex steroid hormone such as estradiol and testosterone in serum as well as spermatocrit, count, viability and motility of sperm in *Clarias gariepinus*. Additionally, it caused degenerative and histological abnormalities in the testis. Though, Minimal variations in the total number of spawning eggs and fertilization rate as a result of parental exposure to microplastics was reported in *D. rerio* [73]. Hence, it is obvious that exposure of microplastics to fish may interfere with the functionality of the HPG axis and reduced the level of FSH, LH and sex steroids, which seriously impact the reproductive health of aquatic animals.

Afreen et al. [2] demonstrated a survey to observe the efficacy of microplastics polystyrene on the female reproductive system. In female mice, exposure to polystyrene microplastics enhanced the likelihood of bigger ovaries having lesser follicles, reduced the counts of embryos and lowered the counts of pregnant female. Additionally, it produced oxidative stress and altered the levels of sex hormones, which may have potentially affect reproduction as well as fertilization. Granulosa cells that are exposed to microplastics underwent pyroptosis and apoptosis as a result of the NLRP3/caspase signaling pathway being activated and Wnt signaling pathway being disrupted. The uterine fibrosis that led to the thinning of the endometrium was brought on by TL4/NOX2 activation. The quality and maturation of the oocyte as well as ovarian capacity were all negatively impacted by the polystyrene microplastics. Additionally, the microplastics interfered with the HPG axis, resulting in a reduction in the body size of the offspring and the rate of hatching, having cross-generational impacts. Similar kind of study should be carried out centralizing on the precise mechanism of action of the microplastics for the dysfunctioning of the reproductive system in fish.

Application of the microplastic to the fish could potentially lead to the apoptosis of the gonadal tissue. Microplastic exposure of polystyrene at 1000 µg/L for 21 days markedly enhanced p53 mediated apoptosis in the gonadal tissue of male *D. rerio*. The histological observation of the gonad suggested marked reduction in the thickness of the basement membrane of testis after exposure to microplastics. Fish gonad that are exposed to microplastics can experience histological changes and molecular responses, which may have a detrimental effectivity on the reproductive organs of fish [72]. Polyethylene microplastics (1 and 10 mg/L) for 15 or 30 days have the ability to bioaccumulate in the gonadal tissue, interfere with the transcription of genes associated with the HPG axis, change the levels of sex hormones, raise pathological lesions in gonad and apoptosis, causing harm to the biological qualities of semen and impact on the reproduction of the parental generation in *Paramisgurnus dabryanus*. Microplastics that are still remain persist in the gonads of parents have the potential to be transferred to the embryos and bioaccumulated on the chorionic extraembryonic membrane, an increase in malformation and mortality rates, increasing the time of hatching and reducing body length and hatching rate. Polyethylene microplastics may have a negative effect on reproduction and have also an important adverse effect on the sustainability of the population. The impacts of microplastics on the teleost fish reproductive injury and cross generational impacts, which have ramifications for ecotoxicology as well as in aquaculture [99]. Sun et al. [89] also suggested that, polystyrene microplastics had several impacts on reproductive dysfunction of male and cross-generational toxicity in the freshwater prawn. Wang et al. [96] argued that parental exposure of polystyrene microplastics (10 µm) at 2, 20 and 200 µg/L for 60 days in *O. melastigma* has an impact on the earlier development of offspring, hence supporting cross-generational role of the microplastics. Conversely, it was argued that though some remarkable changes such as mRNA expression of sex steroids was observed in the parents of *D. rerio*, but exposure of polystyrene microplastics in parental generation may have minimal or reversible cross-generational impact [73]. Finally, it can be said that exposure of microplastics caused apoptosis to the gonadal tissue and can have the potential to exert cross-generational impact, though further research should be required to confirm their cross-generational effect in fish.

Growth, differentiation, meiosis and the inclusion of the macromolecules required for the developing embryo such as proteins, carbohydrates, lipids, vitamins, hormones, growth factors, transcription factors and enzymes, all occur during oogenesis [14]. Growth and maturation phases are the two distinct phases of oogenesis, where the growth phase or vitellogenesis is controlled by FSH. In all oviparous vertebrates, the liver produces and secretes vitellogenin at the time of vitellogenesis. Vitellogenin is a complex as well as bulky phosphorous-glycoprotein that reacts to the estradiol produced by the follicular cells by binding to calcium [30]. The liver produces vitellogenin, which is then discharged into the circulation. The vitellogenin is then sequestered by the developing oocytes via specific receptors arranged in clathrin-coated pits, causing vitellogenin-coated vesicles to form and penetrate the peripheral oolema [40]. It has been shown that microplastic may suppress the transcription of GnRH in the hypothalamus of *C. gariepinus* as well as choriogenin and vitellogenin in the liver of *Oryzias latipes* [77], [52]. Exposure of polystyrene microplastics (1 µm) at 0.01, 0.1 and 1 mg/L for 14 days reduced the levels of GnRH and vitellogenin of *O. niloticus* [20]. Polystyrene microplastics (10 µm) at 2, 20 and 200 µg/L for 60 days reduced fecundity and slowed the gonadal maturation in the females of *O. melastigma*. Transcriptomic study revealed that exposure of microplastics had a substantial negative regulatory impact on the HPG axis of the females. The expression of the genes associated with the process of steroidogenesis was reduced, hence serum concentration of testosterone as well as 17β-estradiol was lowered. Additionally, parental exposure with microplastics at 20 µg/L concentration delayed the body length, heart rate and hatching rate of *O. melastigma*. GnRH is a physiological regulator of GTH release in the HPG axis, which controls gametogenesis and steroidogenesis in vertebrates. Microplastic may interfere with reproduction, as evidenced by the downregulation of the choriogenin, vitellogenin and GnRH. The gonadal development and role depend heavily on the sex steroids testosterone and 17β-estradiol. The liver receives 17β-estradiol, which is produced in the ovaries and causes the generation of choriogenin and vitellogenin, both are necessary for oogenesis. It is recommended that, fish exposed to microplastics had considerably lower plasma 17β-estradiol levels and transcripts of liver choriogenin and vitellogenin, which may be the primary cause of the retarded ovarian development and lowered GSI value. When female *O. melastigma* were exposed to 2, 20 and 200 µg/L of microplastics, downregulation of the genes, including LHR and FSHR in the ovary and mGnRH, LHβ, FSHβ and GTHα in the brain were observed, and these data revealed that microplastics had a detrimental regulatory effect on the HPG axis, which caused the levels of 17β-estradiol to drop. Exposure to microplastics inhibited the steroidogenesis pathway in females by downregulating 11βHSD, 17βHSD, StAR, CYP11α2 and CYP17α1. Given the fact that steroidogenesis pathway is in charge of producing sex steroids, exposure to microplastics inhibited the synthesis of sex hormones, which in turn reduced their levels in the plasma of females. On contrary, male *O. melastigma* exposed to microplastics showed slightly higher levels of testosterone and 17β-estradiol and elevated transcription of genes implicated in the steroidogenesis pathway and HPG axis. Thatswhy, authors concluded that the endocrine disruption caused by polystyrene microplastics was sex-specific. In short, microplastics disrupted the steroidogenesis and HPG axis, delaying ovarian development, and upsetting the balance of sex steroids and also decreased the production of choriogenin and vitellogenin in the liver of female *O. melastigma* [96].

The reproductive system of the living animals are disrupted by polystyrene microplastics, which are often regarded as endocrine disruptors. The reproductive endocrinopathy known as polycystic ovarian syndrome (PCOS) has long been a cause of concern because of its lasting effects on infertility as well as reproductive dysfunction. There is little research on the relationship between daily consumption of polystyrene microplastics through drinking water and the development of PCOS and inducing ovarian fibrosis in long-term exposure, despite multiple findings on endocrine and reproductive toxicity. Polystyrene microplastics consumed daily demonstrated the classic pathophysiology of PCOS in *D. rerio viz.* increased percent GSI and body weight, reduced 17β-estradiol and FSH, enhanced LH and ovarian as well as brain testosterone level. In accordance, histology of the ovary exhibited cystic lesions such as oocyte buddings, hypertrophy of theca, invagination of zona pellucida, disorganization of the follicular membrane and basophilic granular buildup, in addition to a greater number of developing (stage I and II) and fewer mature oocytes. There was a considerable disruption in the expressivity of the biomarkers associated with PCOS, including fem1a, dennd1a and tox3. Microplastics have a major effect in the development of PCOS by increasing the oxidative stress, which in turn induces inflammation and exacerbates ovarian mitophagy, showing how it might exacerbate PCOS into fibrosis of the ovarian tissue, a condition marked by the accumulation of collagen and the overexpression of genes that are pro-fibrogenic markers [1]. In short, microplastics may generate PCOS, affect the HPG axis of the fish, modulate the process of steroidogenesis and apoptosis of the gonadal tissue, hence causes reproductive dysfunction in fish species.

## 4 Conclusion

After being exposed, microplastics mostly bioaccumulated in guts and gills of fish, then, through the use of circulation, the particles spread to and bioaccumulated in other crucial tissues. The degree of bioaccumulation is determined by the size of the microplastics rather than the routes of exposure *e.g.* dietary vs. waterborne or ecosystem either salt or freshwater. The circulatory system of fish is impacted by the microplastic bioaccumulation in their tissues, influencing a number of hematological indices that are connected with immune response, osmotic pressure, clotting of blood, molecular transport and metabolism of fat. Fish exposed to microplastics have different levels of ROS generation depending on whether antioxidant responses are stimulated or inhibited, as well as glutathione and its associated pathways are also disturbed, eventually causes oxidative injury by upsetting the antioxidant homeostasis. Furthermore, fish that are exposed to microplastics experienced immunological toxicity, which either stimulated or inhibited the immune responses. Additionally, fish exposed to microplastic had reduced level of acetylcholine esterase, which resulted in cognitive and behavioral disorders. Exposure to microplastics caused hepatic and gut tissue injury, affect barrier function and dysbiosis of the microbial composition of the gut, alter the metabolism of host, affecting the activity of the digestive enzymes, eventually affecting the growth performance of fish. Microplastics exposure target the HPG axis and interfere with the process of steroidogenesis, apoptosis of the gonadal tissue, ultimately causing reproductive dysfunction. In summary, fish exposed to microplastics have a range of toxic effects *viz.* alteration to immune, antioxidant and hematological indices, bioaccumulation as well as neurotoxicity, growth inhibition and reproductive dysfunction. It was established that the size of the microplastic particles as well as their dose played a significant role in generating toxic effects.

## Declarations

Ethics approval and consent to participate not applicable.

## Consent for publication

Not applicable.

## Ethics statement

For this review paper, neither human nor animal testing was conducted.

## Authors’ contributions

The sole author are responsible for conceptualization, validation, data curation, data compilation, draft writing, editing, supervision, review and submission of the manuscript.

## Funding

This review article is not supported by any funds.

## Declaration of Competing Interest

The authors declare that they have no known competing financial interests or personal relationships that could have appeared to influence the work reported in this paper.

## Data Availability

The authors do not have permission to share data.
